# Identification and genomic analysis of temperate *Halomonas* bacteriophage vB_HmeY_H4907 from the surface sediment of the Mariana Trench at a depth of 8,900 m

**DOI:** 10.1128/spectrum.01912-23

**Published:** 2023-09-20

**Authors:** Yue Su, Wenjing Zhang, Yantao Liang, Hongmin Wang, Yundan Liu, Kaiyang Zheng, Ziqi Liu, Hao Yu, Linyi Ren, Hongbing Shao, Yeong Yik Sung, Wen Jye Mok, Li Lian Wong, Yu-Zhong Zhang, Andrew McMinn, Min Wang

**Affiliations:** 1 College of Marine Life Sciences, Institute of Evolution and Marine Biodiversity, Frontiers Science Center for Deep Ocean, Center for Ocean Carbon Neutrality, Ocean University of China, Qingdao, China; 2 School of Oceanography, Shanghai Jiao Tong University, Shanghai, China; 3 UMT-OUC Joint Academic Centre for Marine Studies, Qingdao, China; 4 Department of Integrated Global Studies, School of Integrated Arts and Sciences, Hiroshima University, Hiroshima, Japan; 5 Institute of Marine Biotechnology, Universiti Malaysia Terengganu, Kuala Terengganu, Malaysia; 6 State Key Laboratory of Microbial Technology, Marine Biotechnology Research Center, Shandong University, Qingdao, China; 7 Institute for Marine and Antarctic Studies, University of Tasmania, Hobart, Tasmania, Australia; 8 Haide College, Ocean University of China, Qingdao, China; 9 The Affiliated Hospital of Qingdao University, Qingdao, China; National Chung Hsing University, Taichung, Taiwan

**Keywords:** temperate phage, *Halomonas*, genomic analysis, Suviridae, hadal sediment, Mariana Trench

## Abstract

Viruses play crucial roles in the ecosystem by modulating the host community structure, mediating biogeochemical cycles, and compensating for the metabolism of host cells. Mariana Trench, the world’s deepest hadal habitat, harbors a variety of unique microorganisms that have adapted to its extreme conditions of low temperatures, high pressure, and nutrient scarcity. However, our knowledge about isolated hadal phage strains in the hadal trench is still limited. This study reported the discovery of a temperate phage, vB_HmeY_H4907, infecting *Halomonas meridiana* H4907, isolated from surface sediment from the Mariana Trench at a depth of 8,900 m. To our best knowledge, it is the deepest isolated siphovirus from the ocean. Its 40,452 bp linear dsDNA genome has 57.64% GC content and 55 open reading frames, and it is highly homologous to its host. Phylogenetic analysis and average nucleotide sequence identification reveal that vB_HmeY_H4907 is separated from the isolated phages and represents a new family, *Suviridae*, with eight predicted proviruses and six uncultured viral genomes. They are widely distributed in the ocean, suggesting a prevalence of this viral family in the deep sea. These findings expand our understanding of the phylogenetic diversity and genomic features of hadal lysogenic phages, provide essential information for further studies of phage-host interactions and evolution, and may reveal new insights into the lysogenic lifestyles of viruses inhabiting the hadal ocean.

**Importance:**

*Halomonas* phage vB_HmeY_H4907 is the deepest isolated siphovirus from the ocean, and it represents a novel abundant viral family in the ocean. This study provides insights into the genomic, phylogenetic, and ecological characteristics of the new viral family, namely, *Suviridae*.

## INTRODUCTION

Viruses are thought to be the most abundant non-cellular structures in the ocean, capable of driving the microbial food loop in marine ecosystems through viral shuttling and viral shunting, influencing the biological carbon pump, adding to the ocean carbon flux, and subsequently influencing climate change; they are essential regulators of global ecology (1 – 4).

The hadal trench is the planet’s least explored and most mysterious environment, and it is the deepest habitat for life on Earth’s surface (5). With significant hydrostatic pressure, below-freezing temperatures, and total darkness, the trench is a somewhat secluded, contained, and very oligotrophic environment (6). The special environment contains a variety of novel prokaryotes with specific metabolic properties (e.g., Thaumarchaeota, Nitrospirae, and Nitrospinae), and hydrocarbon-degrading microorganisms are present in great abundance (7 – 9). Recently, metagenomes have been applied in viral investigations in the hadal trenches such as the Japan Trench, Izu-Ogasawara Trench, Mariana Trench, and Izumi Trench as deep-sea sampling methods have advanced (10 – 14). Jian concluded that there is some exchangeability among trench viruses and suggested that hadal viruses (mainly phages) have unprecedented genomic novelty and are ecologically important (12). Zhou et al. suggested that Challenger Deep viruses could regulate carbohydrate and sulfur metabolism in their potential hosts and stabilize host cell membranes under extreme hydrostatic stress (15). Zhao et al. concluded that novel viral communities may contribute to carbon, nitrogen, and sulfur metabolism in the upper slope sediments of the Marianas Trench (13). Gao et al. concluded that viruses in the deep and hadal zones tend to be lysogenic and potentially mediate horizontal gene transfer (14). However, only two strains were isolated from the hadal environments. PstS-1, a lysogenic *Pseudomonas* phage, was induced from 7,000-m-deep seawater in the Japan Trench (16). And HMP1, a temperate phage from *Halomonas* sp. MT08-1, was induced from sediments at 8,636 m in the Mariana Trench (17). Our understanding of hadal viruses has been greatly limited by the scarcity of isolated viruses in the hadal trenches.

The gram-negative halophilic gammaproteobacterium *Halomonas* is a member of the Oceanospirillales order and can be found in various habitats, including the Antarctic, Marianas Trench, and deep-sea hydrothermal vents (7, 18, 19). They were also widely used in synthetic biology because members of *Halomonas* are well known for their capacity to break down petroleum hydrocarbons, flourish in environments with high salt concentrations and alkaline pH, and have a high tolerance to contamination (20 – 22). It is also abundant in the Mariana Trench, suggesting that it may play an essential role in hadal environments (7). Despite its significance, very little is known about the viruses that infect *Halomonas*, with only two phages published in the NCBI data set so far. One is a virulent virus QHHSV-1 (23) isolated from *Halomonas ventosae* QH52-2 from the Qiaohou salt mine in Yunnan, and the other is a myovirus temperate phage HAP-1 that was isolated and induced from surface water in the Gulf of Mexico (24).

In this study, a novel siphovirus infecting *Halomonas*, vB_HmeY_H4907, was isolated from sediments in the Mariana Trench (at a depth of 8,900 m), the deepest known phage isolated to date. Genomic and phylogenetic characteristics of vB_HmeY_H4907 indicate that vB_HmeY_H4907 is different from the isolated phages and clusters with our predicted eight proviruses in the bacterial genomes and six uncultured viral genomes (UViGs) with a high degree of genomic similarity. Therefore, we propose a new viral cluster (VC) at the family level, named *Suviridae*. The ecological distribution of members of *Suviridae* suggests the prevalence of this viral family in the deep sea.

## RESULTS AND DISCUSSION

### Morphology and biological characterization of vB_HmeY_H4907

**FIG 1:**
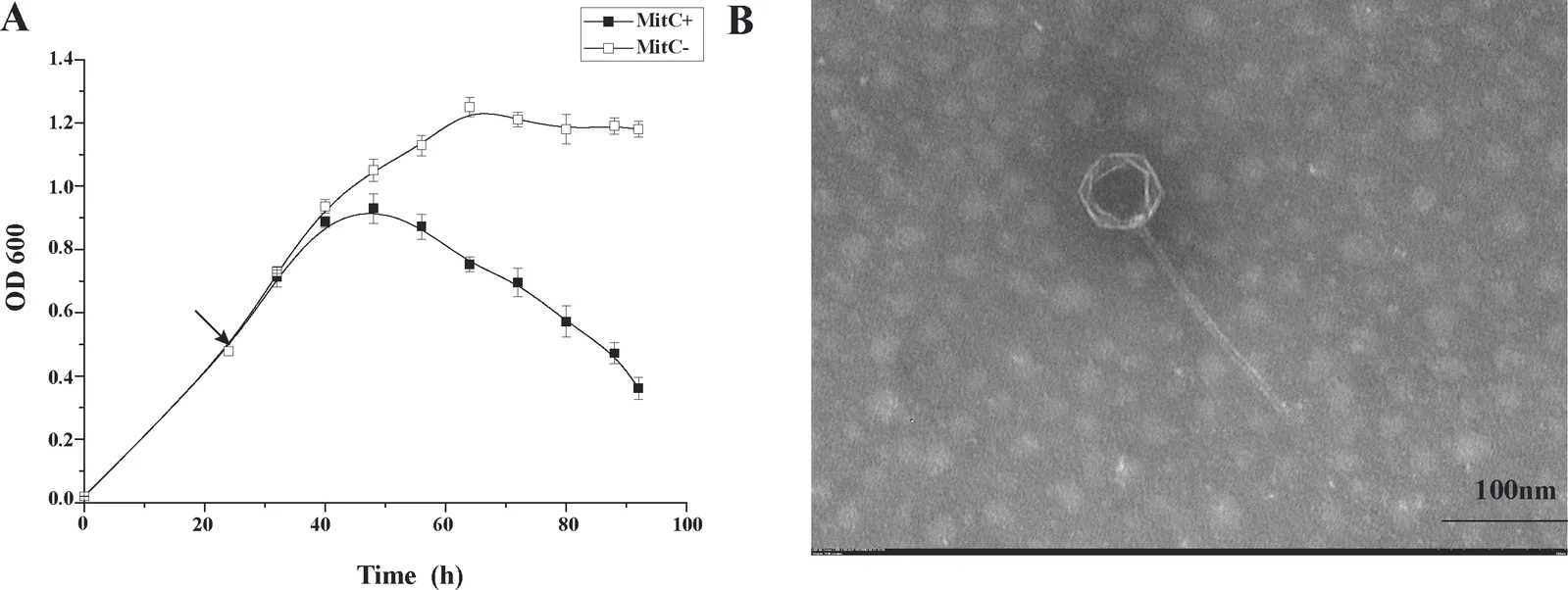
(**A**) Effects of MMC treatment on the growth of *Halomonas* sp. H4907. In the prophage induction experiment, one subculture (solid square) was treated with MMC, while the other subculture (hollow square) served as a control. Bacterium growth was detected at OD_600_, and the time points at which MMC was added are indicated by an arrow. The data shown are average values from triplicate experiments, and error bars indicate standard deviations. (**B**) TEM morphology of *Halomonas* phage vB_HmeY_H4907; the scale bar is 100 nm.

*Halomonas* sp. H4907 was isolated from 8,900-m-deep sediment in the Mariana Trench of the Western Pacific. The 16S rRNA sequence of the host strain was most similar to that of *Halomonas meridiana* (Fig. S1). To examine whether H4907 contained inducible temperate phage, mitomycin C (MMC) (1 µg/mL) was added to H4907 cultures at the early stage of the exponential growth period (24 h, OD_600_ = 0.5). We found that the growth of H4907 was significantly inhibited by the addition of MMC, suggesting that bacterial lysis induced by prophage may have occurred (Fig. 1A). Unlike the untreated group, the host bacterial suspension became clear after incubation with *Halomonas* phage vB_HmeY_H4907 (about 96 h), which indicated that the bacteria were killed by the phage (Fig. S2). TEM reveals the siphoviral morphology of *Halomonas* phage vB_HmeY_H4907, which has an icosahedron head (average diameter: 65 nm) and a long, non-contractible tail (average length: 183 nm) (Fig. 1B). The phage vB_HmeY_H4907 was a temperate phage induced by MMC, and the integrase was also present on the genome, but it did not show obvious phage plaques. However, there was a clear clarification of the host bacterial suspension in the liquid infection, so we hypothesized that there might be a phage re-infection of the host bacterium.

### Genomic features of phage vB_HmeY_H4907

According to the sequencing and assembly data, the linear dsDNA genome length of vB_HmeY_H4907 was 40,452 bp, with a GC content of 57.64%. By the analysis of GeneMarkS (25), RAST (26), Glimmer (v3.02) (27), and Prodigal (Prokaryotic Dynamic Programming Genefinding Algorithm) (V2.6.3) (28), a total of 55 open reading frames (ORFs) of vB_HmeY_H4907 were predicted. The result of the cumulative GC skew analysis indicates that the origin and terminus of replication were at the regions 40,400 nt with the lowest value and 200 nt with the highest value, respectively (29) (Fig. S3).

**FIG 2:**
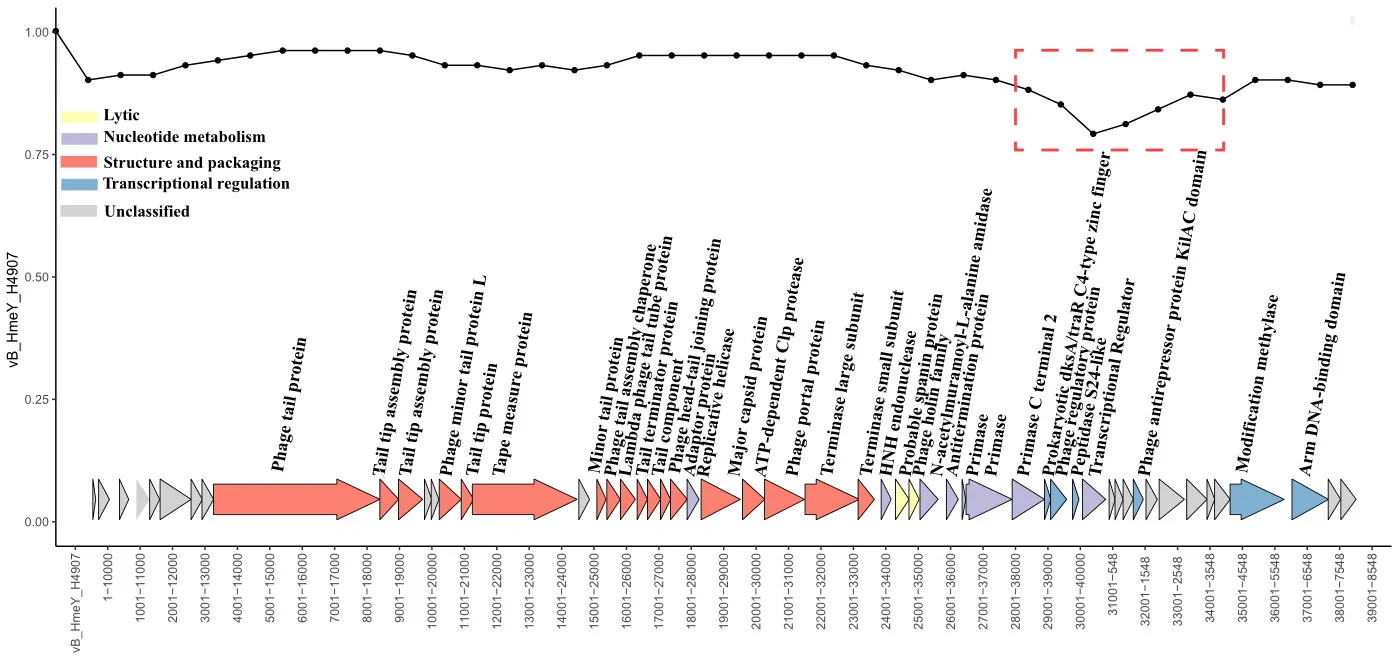
Genome map of the genome sequence of *Halomonas* phage vB_HmeY_H4907. The gene map represents different categories of putative functional genes, represented by different colors. The number is a tetranucleotide correlation. The weaker correlations are circled by a red ellipse.

Out of the 55 coding DNA sequences, 2 of the coding regions (CDS) show no similarity with any known sequences (*E*-value >1e−5), while the whole 55 CDS were categorized into 5 functional modules: lytic, nucleotide metabolism, structure and packaging, transcriptional regulation, and unclassified, based on their predicted known functions (Fig. 2; Table S1).

The vB_HmeY_H4907 genome was characterized by most of its CDS being connected to structure and packaging, predominantly located in the genome’s midsection. However, the CDS associated with the unknown were mainly situated at the beginning of the genome and were homologous to the host, which may imply the mechanism of association between the lysogenic virus and the host. This may be because there are relatively few studies on deep-sea viruses, which further illustrates the significance of this study. Notably, the number of vB_HmeY_H4907 tail-associated proteins was unexpectedly higher than another siphovirus infecting *Halomonas* (2 of the 69 ORFs in *Halomonas* phage QHHSV-1 genomes [23]), with a total of 10 out of 33 proteins with known activities identified as tail-associated proteins. Three tail tip proteins (ORFs 10, 11, and 15), a baseplate hub protein (ORF 9), a tape measure protein (ORF 16), two minor tail proteins (ORFs 14 and 18), a tail tube protein (ORF 20), a phage tail assembly chaperone (ORF 19), and a tail terminator protein (ORF 21) were among the 10 probable tail proteins encoded by the vB_HmeY_H4907 genome (Fig. 2; Table S1). The tail tip complex (30), which is assembled sequentially by three tail tip proteins J, one tail tip protein I (ORF 10), one tail tip protein L (ORF 14), and one tail tip protein K (ORF 11), is completed by adding tail tip protein M (ORF 15) (31) and then interacting with tape measure protein (ORF 16) to initiate tail tube assembly. It is noteworthy that ORF 19 matches tail assembly chaperones (TACs), which are necessary for the morphogenesis of all siphoviruses. TACs wrap around tail-tube proteins to prevent them from creating ineffective complexes or precipitating before incorporating tail-tube proteins (32, 33), which is consistent with the tail shape of vB_HmeY_H4907. ORF 20 is homologous to the phage tail tube protein from the siphovirus of *Gammaproteobacteria*. With the aid of a tail tip complex, a tape measure protein, and two chaperones, it can polymerize and supply host infectivity (33). ORF 21 matches the tail terminator protein, which functions as a gene transfer agent (GTA) that mediates horizontal gene exchange, expelling DNA into the bacterial periplasm through the RcGTA’s restricted hole in the GTA substrate (34). The tails of siphoviruses are exact mechanisms that play a critical role in host cell wall recognition, attachment, and penetration, allowing for efficient delivery of genomic DNA to the host cytoplasm as well as identification of phage-specific characteristics such as host range tactics (29, 35). In the vB_HmeY_H4907 genome, numerous and diverse tail-associated genes are essential for forming tail structures and host interactions.

The lytic module of vB_HmeY_H4907 comprises only two ORFs. P51770 is a member of the spanin complex, homologous to ORF 32, and plays a crucial role in disrupting the host outer membrane during viral efflux and causing cell lysis (36). The spanin complex completes host lysis by rupturing the outer membrane after the actions of holin and endolysin penetrate the inner membrane and break down the host peptidoglycan. ORF 33 was annotated to holins, the product of the lambda phage S gene, which can interact with the host cell membrane to let lytic enzymes penetrate the bacterial cell wall (37). These findings suggest that vB_HmeY_H4907 was similar to the lambda phage in terms of the lysis plate.

The nucleic acid metabolism panel contains seven ORFs, including replicative helicase (ORF 25), HNH endonuclease (ORF 31), N-acetylmuramoyl-L-alanine amidase (ORF 34), antitermination protein (ORF 35), primase (ORF 37), primase C terminal 2 (ORF 38), and peptidase S24-like (ORF 42). ORF 42 encodes the peptidase S24-like and shows homology to the lambda phage (38). ORF 31 was homologous to the endonuclease of the deep-sea thermophilic phage GVE2 (39), and ORF 37 was similar to the phage NrS-1 (40) polymerase that infects *Epsilonproteobacteria*, a major producer of primary productivity in deep-sea hydrothermal vent ecosystems, which may be partly due to the similarity in nucleic acid metabolism of deep-sea phages.

The transcriptional regulation panel contains six ORFs, including prokaryotic dksA/traR C4-type zinc finger (ORF 39), phage regulatory protein CII (ORF 40), transcriptional regulator (ORF 41), phage antirepressor protein KilAC domain (ORF 46), modification methylase (ORF 52), and arm DNA-binding domain (ORF 53). ORF 46 is associated with known phage antirepressors in the KilAC domain (e.g., Ant1 and Ant2 of *Escherichia coli* phages 1 and 7 [P1 and P7]) (41, 42), and the ANT/KilAC domain is responsible for the inhibition of the SOS response, as its overexpression effectively prevented bacterial lysis and SOS gene transcription, which suggested intimate interaction between vB_HmeY_H4907 and the host (43). Since vB_HmeY_H4907 is a temperate phage, the proteins that promote lysogenic infection are highlighted here. Phage regulatory protein CII, DNA methylation-modifying enzyme, and integrase are all present to demonstrate the lysogenic infection of vB_HmeY_H4907. Phage regulatory protein CII consists of several phage regulatory protein CII (CP76) sequences involved in lysogenesis (44, 45). ORF 52 corresponded to a DNA methylation-modifying enzyme, and viral DNA methylation can aid in immunizing the host’s non-specific recognition system, thereby reducing the likelihood of detection by the host as foreign genetic material and promoting lysogenic infection (46). Integrases are essential for the site-specific recombination process that incorporates phages into the host DNA and are also required for removing phages from the host genome, in addition to excision enzymes. The fact that ORF 53 was the integrase of vB_HmeY_H4907 indicates that it can spread temperately in natural environments.

Since there are many ORFs with unknown functions in the vB_HmeY_H4907 phage genome, it is believed that with the continuous improvement of the protein function database and further isolation of abyssal phages, some interesting AMGs will be discovered.

### The tetranucleotide frequency correlation analysis of phage vB_HmeY_H4907

The tetranucleotide correlation between each 10 kb genomic fragment and the entire genome of vB_HmeY_H4907 was shown. Notably, the 13 ORFs (from positions 39 to 51) are much less correlated to tetranucleotides than the rest of the sequence, which suggests that the sequence needs to be better adapted to the genome. The tetranucleotide frequency correlation is lowest (0.79) in ORF 41 (Fig. 2) related to transcriptional regulation, providing further evidence that this module may have a closer relationship with the host genome (47). Importantly, the module with low tetranucleotide frequency correlation is not homologous to proteins in other viruses (*E*-value <1e−5, query cover >50%, and identity >50%), but it can match a large number of homologous proteins in the bacterial genome, indicating a high likelihood of external gene insertion and potential horizontal gene transfer (29). A recent study concluded that viruses in the deep and hadal zones tend to be lysogenic and potentially mediate horizontal gene transfer (14). Perhaps these exogenously inserted modules play a role in adapting to lysogenic infection in the hadal, which is what we need to explore next.

### Phage vB_HmeY_H4907 represents a novel viral cluster

**Fig 3:**
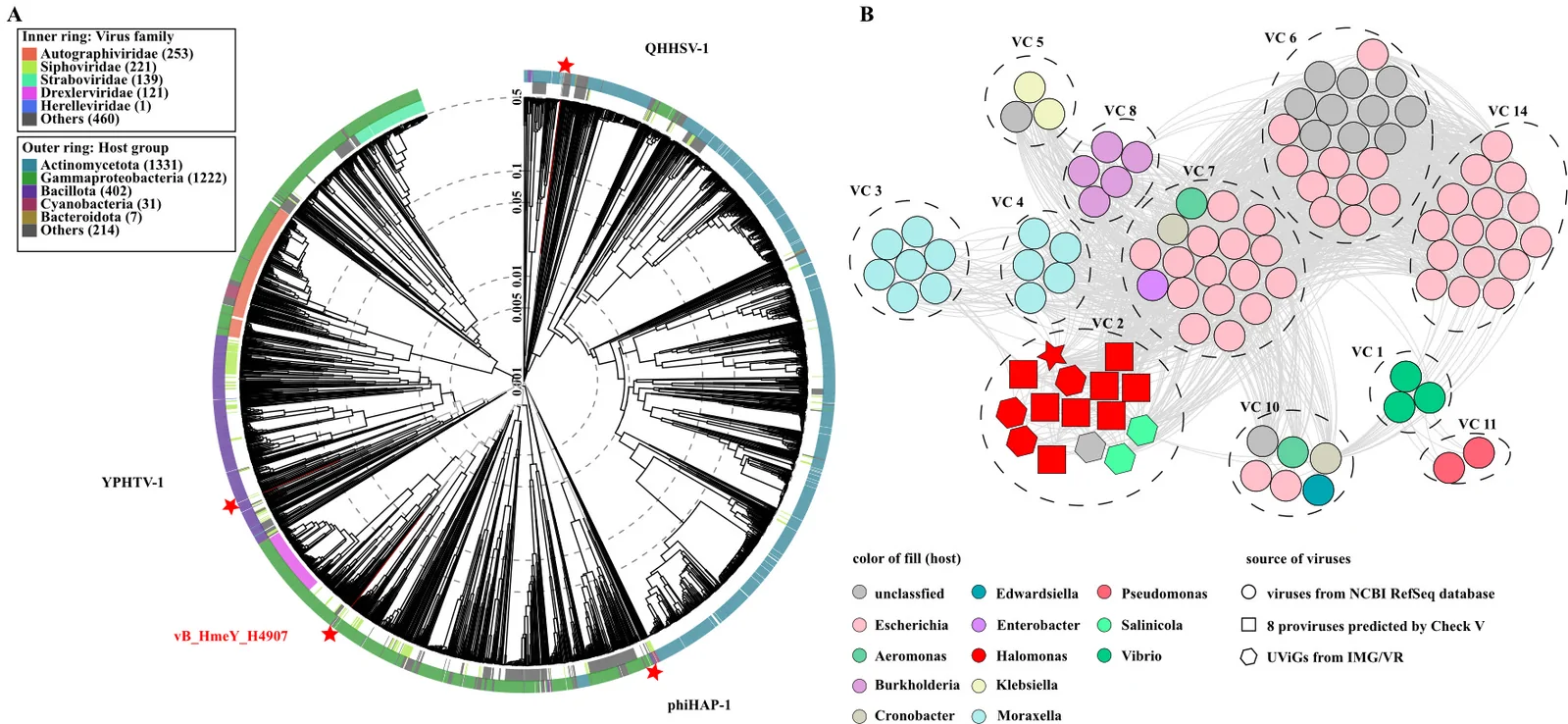
(**A**) Phylogenetic tree of all isolated *Halomonas* phages by VipTree. *Halomonas* phages are in red. (**B**) Gene content-based viral networks showing the viruses from the NCBI RefSeq database, six UViGs related to vB_HmeY_H4907 from IMG/VR, and eight proviruses predicted by CheckV. The nodes represent the viral genomic sequences. The edges represent the similarity scores between genomes based on shared gene content. The related UViGs from IMG/VR are indicated by regular hexagons, and the eight proviruses predicted by CheckV are represented by squares. Among those, the red star represents *Halomonas* phage vB_HmeY_H4907. Viral genomes that are infected by different hosts are indicated by different colors.

The comparative genomic analysis within the *Halomonas* phage group is challenging due to the lack of known sequenced *Halomonas* phages and their spread across other phage families (Fig. 3A). In this study, we utilized BLASTX to search the NCBI database for sequences related to vB_HmeY_H4907 (*E*-value 1e−5) and obtained 169 complete sequences. Subsequently, 10 uncultivated viral genomes that had at least 22 proteins (40% of the ORFs) that were homologous with vB_HmeY_H4907 were selected in the IMG/VR (Integrated Microbial Genomes/Virus) protein database. With the combination of 19 phages against the Oceanospirillales, 10 non-redundant homologous UViGs, 169 phages related to vB_HmeY_H4907 retrieved from the NCBI database, and vB_HmeY_H4907, a total of 189 sequences were collected. And they were then input to vConTACT2 for pre-experiment clustering analysis. The phages connected to vB_HmeY_H4907 in the network diagram of the pre-experiment were searched, and Orthofinder was used to identify their shared unique proteins. A total of eight conserved proteins were obtained (ORF 35, ORF 39, ORF 40, ORF 43, ORF 45, ORF 46, ORF 47, ORF 48). The NR library searched for and downloaded sequences closely linked to the above-mentioned shared proteins in vB_HmeY_H4907. Given that vB_HmeY_H4907 is a lysogenic phage and these sequences are host sequences, CheckV software was used to anticipate the presence of proviruses. After manual checking and removing redundancy, eight proviruses were obtained.

All the mentioned phages were removed from redundancy; a total of 196 phages were analyzed using vConTACT2 for clustering. Among them, vB_HmeY_H4907, six UViGs in the IMG/VR database, and eight proviruses predicted in the NR database were categorized as belonging to virus cluster 2 (VC_2) (Fig. 3B). VC_2 was a relatively independent cluster of viruses that was distinct from other recognized taxonomic clusters, and the majority of clusters are consistent with the taxonomic status of the host. Most viral hosts in VC_2 were classified at the genus level as *Halomonas*, with two viral hosts classified as Salinicola and only one UViG remaining unclassified. And the genus *Halomonas* and the genus *Salinicola* belong to the family *Halomonadaceae*. While the vB_HmeY_H4907 did not cluster with viruses isolated from *Halomonas*, the viruses in VC2 are all proviruses. Therefore, we assume that VC2 may represent a novel phylogenetic branch distinct from known isolated phages from the *Halomonas* genus.

**FIG 4:**
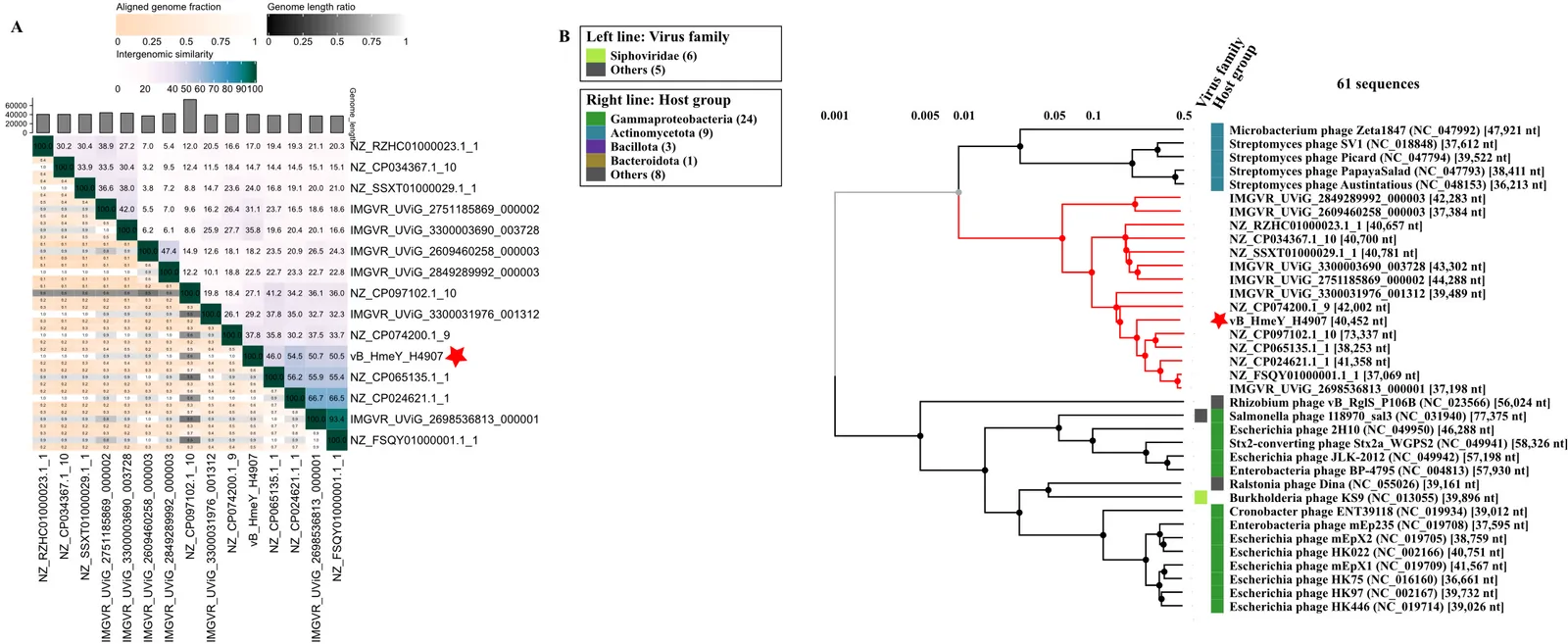
(A) Shared gene heat map of *Halomonas* phage vB_HmeY_H4907 and the 14 closest virus genomes in the rectangular phylogenetic tree. In the right half, color coding allows rapid visualization of phage genome clustering based on intergenic similarity: the closer the relationship between genomes, the darker the color. These numbers represent the similarity values of each genome pair. In the left half, the three indicator values of each genome pair are expressed in order from top to bottom: aligned partial genome 1 (for the genome found in this row), genome length ratio (for two genomes in this pair), and aligned partial genome 2 (for the genome found in this column). Darker colors emphasize lower values, indicating that there is only a small number of genome-aligned genome pairs (orange to white gradient) or a large difference in the length of the two genomes (black to white gradient). As the distance between phages increases, the aligned genomic fragments are expected to decrease. (**B**) Whole-genome-based phylogenetic tree of *Halomonas* phage vB_HmeY_H4907 and the 14 closest virus genomes. The members in VC_2 are shown in red.

To confirm this hypothesis, the sequences of all phages in VC_2 were entered into ViPTree for phylogenetic analysis. The results indicated that the phage-encoded protein in VC_2 had a lower correlation with proteins from other phages, forming a separate branch away from other sequences, possibly representing a new viral cluster (Fig. 4B; Fig. S4). The inter-nucleotide genomic similarity of the 15 phages in VC2 was assessed using VIRIDIC to evaluate this theory at the nucleic acid level, and the results revealed low (14.7%–54.5%) inter-nucleotide genomic similarity between vB_HmeY_H4907 and other phages (Fig. 4A). The phage VB_HmeY_H4907 most likely represented a novel family under Caudoviricetes viruses, according to ICTV, which classifies phages with >70% nucleotide sequence identity as belonging to the same genus (48, 49). Therefore, the phage vB_HmeY_H4907 constitutes a novel family of Caudoviricetes, which we will refer to as the *Suviridae*.

Specific protein sequences’ existence or absence can support the taxonomic foundation of the *Suviridae* and aid in determining the taxonomic status of unclassified *Suviridae*. To further explore the relationship between vB_HmeY_H4907 and other members of the *Suviridae*, protein numbers for vB_HmeY_H4907 and related viruses in the same viral cluster were extracted based on the vConTACT2 results and represented by different protein cluster (PC) values. A heat map was generated using an R package to perform protein clustering network analysis, as shown in Fig. S6. According to the heat map, vB_HmeY_H4907 and its associated genome are split into 15 distinct VCs, each containing different PCs. Among them, *Suviridae* clustered as VC_2, which contains 15 PCs, 4 of which are specific to VC_2, including PC0095, PC0099, PC0101, and PC0109, reflecting the specificity of *Suviridae* phages and further validating *Suviridae*’s taxonomic status. Most phages in the *Suviridae* family include PC0051, PC0090, PC0119, PC0133, and PC0153, while just a few phages lack them (Fig. S3). Notably, the majority of PCs found in the *Suviridae* are located at protein loci determined by vB_HmeY_H4907 tetranucleotide analysis with high host relevance (ORF 39–51) compared to other PCs, reflecting the high homology of the vB_HmeY_H4907 phage with the host and possibly also the high homology of the *Suviridae* phage with the host. This may support the idea that lysogenic phages have co-evolved with their hosts, given that all members of the *Suviridae* share a lysogenic lifestyle.

Considering the difficulty of isolating phages from the deep sea, there are fewer studies on isolating deep-sea phages. The vB_HmeY_H4907 is far from currently known isolated phages but forms a family with uncultured phages in the database. However, it is not clear whether this is because there are fewer phages isolated from this environment or because of the novelty of the protein or system of this phage, which causes vB_HmeY_H4907 to be distantly related to known isolated phages. And some ORFs of vB_HmeY_H4907 are unknown, and the isolation of phages from the hadal is still needed in the future to further expand the database and provide a theoretical basis for our exploration of survival strategies in deep-sea viruses.

### Ecological distribution of vB_HmeY_H4907 in the ocean

**FIG 5:**
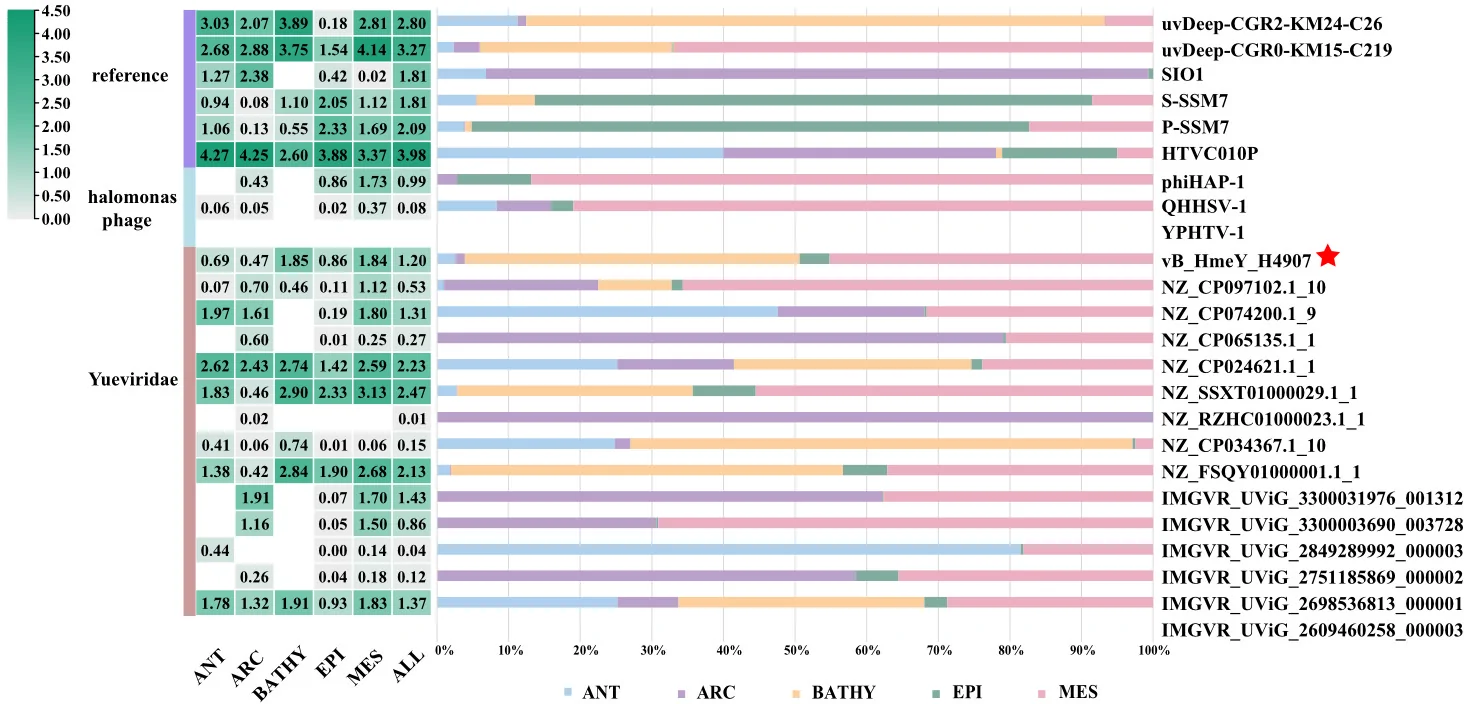
Relative abundances of *Halomonas* phage vB_HmeY_H4907 compared to the abundances of isolated and uncultured phages of high abundance in the ocean and other *Halomonas* phages in the 154 viromes of the GOV 2.0 data set. Relative abundances, expressed by RPKM (reads per kilobase per million mapped reads) values, were calculated using the metagenomics tool minimap2. Left, relative abundances of different phages in five marine VEZs defined by GOV 2.0. Values were normalized by the number of databases in each VEZ, and results were log10 transformed for description. Right, distributions of phages in five VEZs are shown as percentages.

In the Global Oceanic Viromes 2 (GOV 2.0) data set, which includes five viral ecological zones (VEZs), including the Arctic (ARC), Antarctic (ANT), bathypelagic (BATHY), temperate and tropical epipelagic (EPI), and mesopelagic (MES), the biogeographic distribution characteristics of vB_HmeY_H4907 and its closely related viral sequences were examined. The virus in the *Suviridae*, three viruses infecting *Halomonas*, two uncultured abundant UViGs in the BATHY, and four isolated phages with high abundance in the ocean were all included in the reference genome (Fig. 5).

The results confirmed that HTVC010P was one of the most prevalent viruses in the ocean. Moreover, uvDeep-CGR0-KM15-C219 and P-SSM7 were both widely distributed.

Despite its moderate quantity in comparison to the great abundance of phages in the ocean, vB_HmeY_H4907 was most prevalent when compared to phages that infected the *Halomonas*, and only vB_HmeY_H4907 was found in five habitats, which is in accordance with the distribution of the *Halomonas*. In the BATHY, vB_HmeY_H4907 was the least common deep-sea phage compared to uncultivated deep-sea phages, but it was second only to HTVC010P in the common isolated marine phages. Notably, the abundance of some phages in *Suviridae* even outpaces that of the highly abundant reference phages in the ocean, demonstrating that prophages were not insignificantly abundant there, which also highlights the importance of this study in shedding light on the prevalence and distribution of prophages, particularly in the newly discovered *Suviridae* family.

### Conclusion

The hadal trench is the deepest habitat on the Earth’s surface and is rich in microorganisms (5). Recent research has revealed the enormous diversity, novelty, and ecological significance of hadal viruses (12 – 14). However, only two strains of hadal viruses have been isolated. In this study, we isolated and introduced the siphovirus phage vB_HmeY_H4907 infecting *Halomonas* at 8,900 m in the Mariana Trench sediment, the deepest known isolated phage. Genomic analysis showed that phage vB_HmeY_H4907 is evolutionarily distant from other reference viruses and forms a separate branch from six UVIGs and eight proviruses with unique conserved protein genes, representing a new family of Caudoviricetes viruses called *Suviridae*. The phage vB_HmeY_H4907 is widely distributed and in high abundance in the ocean, further demonstrating the lysogenic life strategy of hadal phages. This study deepens our understanding of hadal phages' genetic diversity and genomic features. The phage vB_HmeY_H4907 is highly homologous to its host. It provides a theoretical basis for an in-depth analysis of the life strategy of viruses in extremely harsh environments and their co-evolution with hosts.

## MATERIALS AND METHODS

### Phage isolation and prophage induction

*Halomonas* sp. H4907 was isolated from 8,900 m of sediment in the Mariana Trench in the Western Pacific (142°09′ E, 11°20′ N). This bacterial strain was provided by Yuzhong Zhang’s laboratory at Shandong University and cultured in 2216E medium (peptone 5 wt.%, yeast extract 1 wt.%) at 28°C. The strain was identified by searching the 16S rRNA gene sequence (50). To test whether *Halomonas* sp. H4907 contained an inducible temperate phage, MMC (1 µg/mL) was added to H4907 cultures at the early stage of the exponential growth period (24 h, OD_600_ = 0.5) (16). The experimental group was incubated at 28°C in a constant-temperature incubator at 8 h intervals to determine whether there was significantly more clarification than in the control group. Prophage induction was considered to have occurred when the solutions of the induced cultures were detected to be significantly clarified compared to the control. This experiment was repeated three times.

### Phage particle purification

To concentrate and purify the phage particles from the induction experiments, the combined solution of virus and host was passed through a 0.22-µm filter membrane to remove cellular debris (17). The lysate was then concentrated from 500 to 5 mL using a 30-kDa ultrafilter (UFC5030; Millipore). The concentrated phage lysate was filtered a second time through a 0.22-mm Supor membrane (51). The supernatant was added to PEG 8000 and NaCl at a final concentration of 10% (wt/vol) and 1 M, respectively, and incubated overnight at 4°C. After precipitation, the pellet was centrifuged at 15,000 × *g*, left for 30 min, and then resuspended in 5 mL of SM buffer (100 mM NaCl, 8 mM MgSO_4_, 50 mM Tris-HCl, pH 7.5) (52). The purified phage solution was stored at 4°C away from light.

### Morphology study by TEM

Transmission electron microscopy (TEM) was used to analyze the vB_HmeY_H4907 morphology. Briefly, 20 µL of the concentrated phage solution was taken in drops on a copper grid, absorbed in the dark for 15 min, and then negatively stained with 2 wt.% tungstate phosphate (pH 7.5) for 5 min. The electron micrographs of vB_HmeY_H4907 were captured by a JEOL Model JEM-1200EX TEM at 100 kV (53).

### DNA extraction and genome sequencing

DNA was extracted from the concentrated phage lysate using a Virus DNA Kit (E.Z.N.A.O Viral DNA Kit) according to the manufacturer’s instructions, and quality control was subsequently carried out on the purified DNA samples. Genomic DNA was quantified using a TBS-380 fluorometer (Turner BioSystems Inc., Sunnyvale, CA, USA). A highly qualified DNA sample (OD_260/280_ = 1.8–2.0, >6 µg) is utilized to construct the fragment library. Sequencing was performed by Shanghai Biozeron Biotechnology Co., Ltd. (Shanghai, China). For Illumina pair-end sequencing of the phage, at least 1 µg of genomic DNA was used for sequencing library construction. Paired-end libraries with insert sizes of ~400 bp were prepared following Illumina’s standard genomic DNA library preparation procedure. Purified genomic DNA is sheared into smaller fragments with a desired size by Covaris, and blunt ends are generated by using T4 DNA polymerase. After adding an “A” base to the 3′ end of the blunt phosphorylated DNA fragments, adapters are ligated to the ends of the DNA fragments. The desired fragments can be purified through gel electrophoresis, selectively enriched, and amplified by PCR. The index tag could be introduced into the adapter at the PCR stage as appropriate, and we did a library quality test. The qualified Illumina pair-end library would be used for Illumina NovaSeq 6000 sequencing (150 bp*2; Shanghai BIOZERON Co., Ltd.). The Illumina sequencing data were quality clipped by Trimmomatic (54) and assembled using ABySS (55), and the contigs obtained by splicing were complemented with GAP using GapCloser (56).

Gene models were identified using GeneMarkS (25), RAST (26), Glimmer (v3.02) (27), and Prodigal (Prokaryotic Dynamic Programming Genefinding Algorithm) (V2.6.3) (28). We manually screened the start and end sites repeated in multiple programs and the sequences with start and stop codons to determine the open reading frame. The conserved domain was detected by finding homologs in the non-redundant database (http://blast.ncbi.nlm.nih.gov/), the InterPro database (57), the Conserved Domain Database Suite (CDD/SPARCLE) (58), the UniProtKB database (36), the Pfam database (http://pfam.xfam.org/), and the HHpred server (59). To identify the start and end of the phage genome’s replication, the cumulative GC skew (https://genskew.csb.univie.ac.at/webskew) analysis was applied (29).

### Search for viral sequences homologous to vB_HmeY_H4907 in NCBI and IMG/VR

The genome sequence of vB_HmeY_H4907 was queried against the NCBI database using BLASTx (http://blast.ncbi.nlm.nih.gov/) (*E*-value 1e−5) to expand the homologous sequences of vB_HmeY_H4907. At the same time, all of the protein sequences of vB_HmeY_H4907 were queried against the whole IMG/VR protein database using BLASTp with an *E*-value of 1 × 10^−5^ to recruit as many homologous sequences as possible. Finally, 10 uncultivated viral genomes that had at least 22 proteins (40% of the ORFs) that were homologous with vB_HmeY_H4907 were selected (60, 61).

### Tetranucleotide correlation analysis

The vB_HmeY_H4907 genome was divided into 41 fragments (10 kb for window size and 1 kb for step size), and the fragments were extended by reverse complementation to analyze the tetranucleotide correlation of the phage (47). A maximal-order Markov model was used to generate the 256 potential tetranucleotide frequencies, and the differences between frequencies were transformed into Z-scores (62). Pearson correlation coefficients for the genome and its parts were determined using the genome as a whole.

### Phylogenetic analysis and comparative genomic analyses

A total of 169 phages related to vB_HmeY_H4907 were retrieved from the NCBI database, and 19 phages that infect the Oceanospirillales were downloaded from NCBI Virus (https://www.ncbi.nlm.nih.gov/labs/virus/vssi/#/) by November 2022. In addition, 10 highly confident phages that were associated with vB_HmeY_H4907 were downloaded from IMG/VR (63). vConTACT2 2.0 was used to identify phages connected to vB_HmeY_H4907 by analyzing protein sharing between phages (64). Next, Orthofinder (65) (version: 2.5.2) was performed to cluster co-conserved proteins at the same viral clusters as vB_HmeY_H4907 through all-to-all BLASTp analysis (*E*-value < 1e−5, Query Cover >50%, and identity >30%). BLASTp (*E*-value 1e−5) was used to search the NR database (non-redundant protein sequence database) for the related protein sequence of the co-conserved protein in vB_HmeY_H4907. Then, the nucleic acid sequences corresponding to the protein sequences were downloaded from the NT database (Nucleotide Sequence Database).

Given that all the nucleic acid sequences associated with the vB_HmeY_H4907 acquired from the NT database were sourced from bacterial genomes, the CheckV (66) v0.7.0 program was used to determine whether they contained proviruses in their genome, and 10 proviruses associated with the vB_HmeY_H4907 were finally yielded after being manually screened.

All of the aforementioned phages, including the provirus predicted by CheckV, were clustered using CD-HIT (67) v4.8.1 (parameters: -c 0.99G 1) to remove redundancy and then used for gene-shared network analysis by vConTACT2 (parameters: *E*-value, 1e−5; alignment region covering more than 50% of the shorter sequence; and identity 30%). The Gephi (68) served as the network diagram’s visualization. PCs were divided using the Markov clustering algorithm, and a clustering heatmap of PCs was created using the “complete” technique. Using the vB_HmeY_H4907 genome in conjunction with 14 related sequences, ViPTree (https://www.genome.jp/viptree) was used to construct a genome-wide phylogenetic tree, and VIRIDIC (69) was used to generate a heat map of genes shared with the sequence.

### Ecological distribution in the ocean

The metagenomics tool CoverM (v0.3.1) (https://github.com/wwood/CoverM) used the following parameters: -m rpkm -min-read-percent-identity 0.95 -min-read-aligned-percent 0.75 to compute the relative abundances of viral genomes in the GOV 2.0 database (https://de.cyverse.org/data/ds/iplant/home/shared/iVirus/GOV2.0?selectedOrder=asc&selectedOrderBy=name&selectedPage=0&selectedRowsPerPage=100) (70). Five VEZs were created by GOV 2.0 by dividing the 154 virome databases into the ARC, ANT, BATHY, EPI, and MES (70). The relative abundance of vB_HmeY_H4907 and other phages infecting *Halomonas* was analyzed to comprehend the dispersion throughout the global ocean, with some representative viruses that widely spread in the ocean (71, 72) as references.

## Data Availability

The complete genome sequence of phage vB_HmeY_H4907 has been stored in GenBank with accession number OQ832096. The 16S rRNA sequence of the host has also been deposited in the NCBI GenBank under accession number OQ874717.

## SUPPLEMENTAL MATERIAL

Fig. S1 spectrum.01912-23-s0001.tif Phylogenetic tree of *Halomonas* H4907 and 50 reference sequences of *Halomonas*.

Fig. S2 spectrum.01912-23-s0002.tif The host strain was incubated with phage vB_HmeY_H4907 suspension for 96 h.

Fig. S3 spectrum.01912-23-s0003.tif Cumulative GC skew analysis of the phage genome sequence.

Fig. S4 spectrum.01912-23-s0004.tif Genomic comparisons between *Halomonas* phage vB_HmeY_H4907 and 14 related viruses.

Fig. S5 spectrum.01912-23-s0005.tif Gene map of *Halomonas* phage vB_HmeY_H4907 and 14 related viruses.

Fig. S6 spectrum.01912-23-s0006.tif Protein cluster analysis between *Halomonas* phage vB_HmeY_H4907 and 14 vB_HmeY_H4907-associated genomes.

Table S1 spectrum.01912-23-s0007.docx Genome annotation of *Halomonas* phage vB_HmeY_H4907.

Table S2 spectrum.01912-23-s0008.docx HHperd hits of *Halomonas* phage vB_HmeY_H4907.

Table S3 spectrum.01912-23-s0009.docx Information of Yueviridae virus in the IMG/VR database.

Table S4 spectrum.01912-23-s0010.docx Information of provirus in Yueviridae by CheckV predicted in the host sequence associated with *Halomonas* phage vB_HmeY_H4907.

ASM does not own the copyrights to Supplemental Material that may be linked to, or accessed through, an article. The authors have granted ASM a non-exclusive, world-wide license to publish the Supplemental Material files. Please contact the corresponding author directly for reuse.
