# Supplementary figures and images for: Identification and genomic analysis of temperate Halomonas bacteriophage vB_HmeY_H4907 from the surface sediment of the Mariana Trench at a depth of 8,900 m
Source: Microbiol Spectr. 2023 Sep 20;11(5):e01912-23. doi: 10.1128/spectrum.01912-23 (PMC10580944; doi:10.1128/spectrum.01912-23)

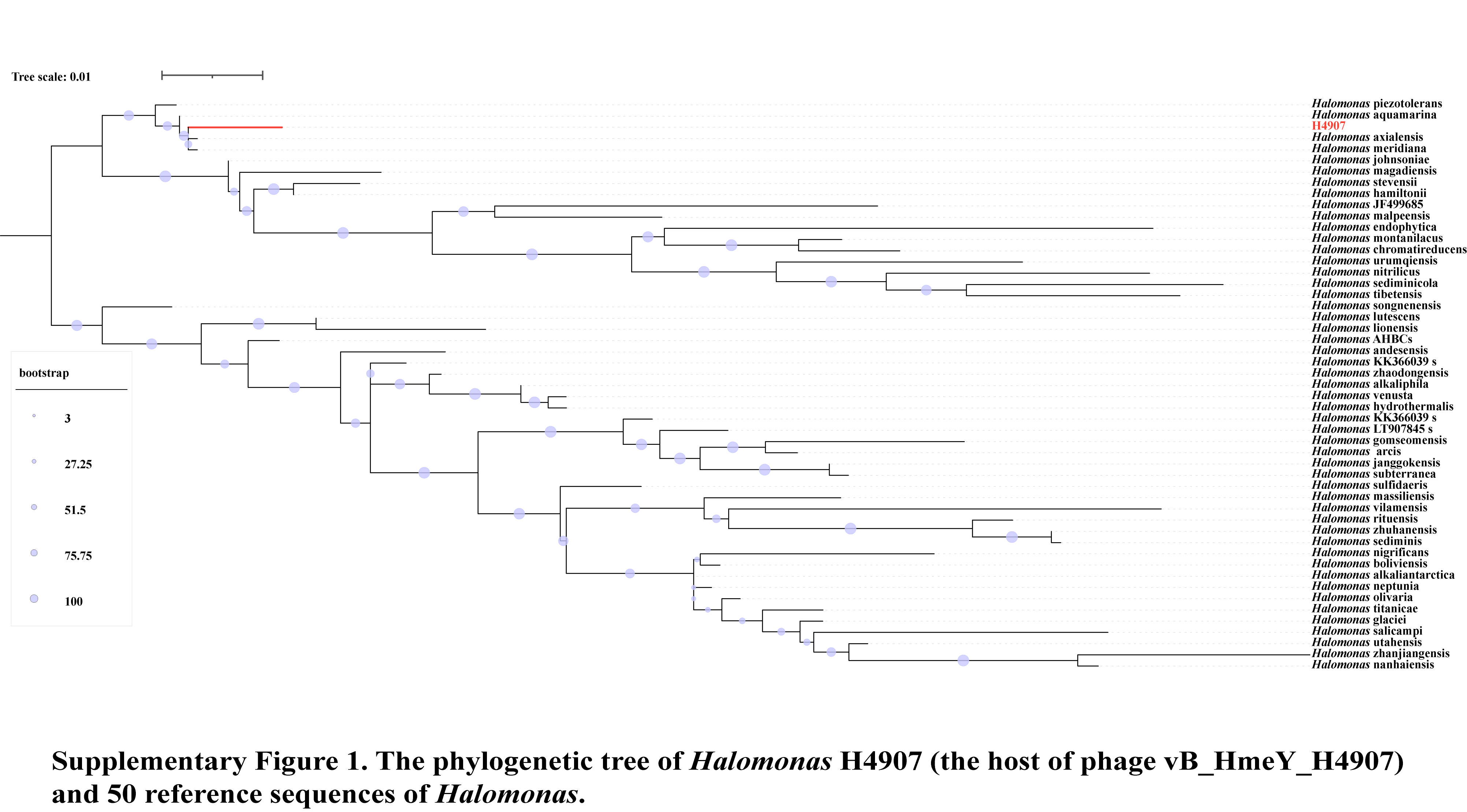

Supplement: Fig. S1 — Phylogenetic tree of Halomonas H4907 and 50 reference sequences of Halomonas. [file spectrum.01912-23-s0001.tif]

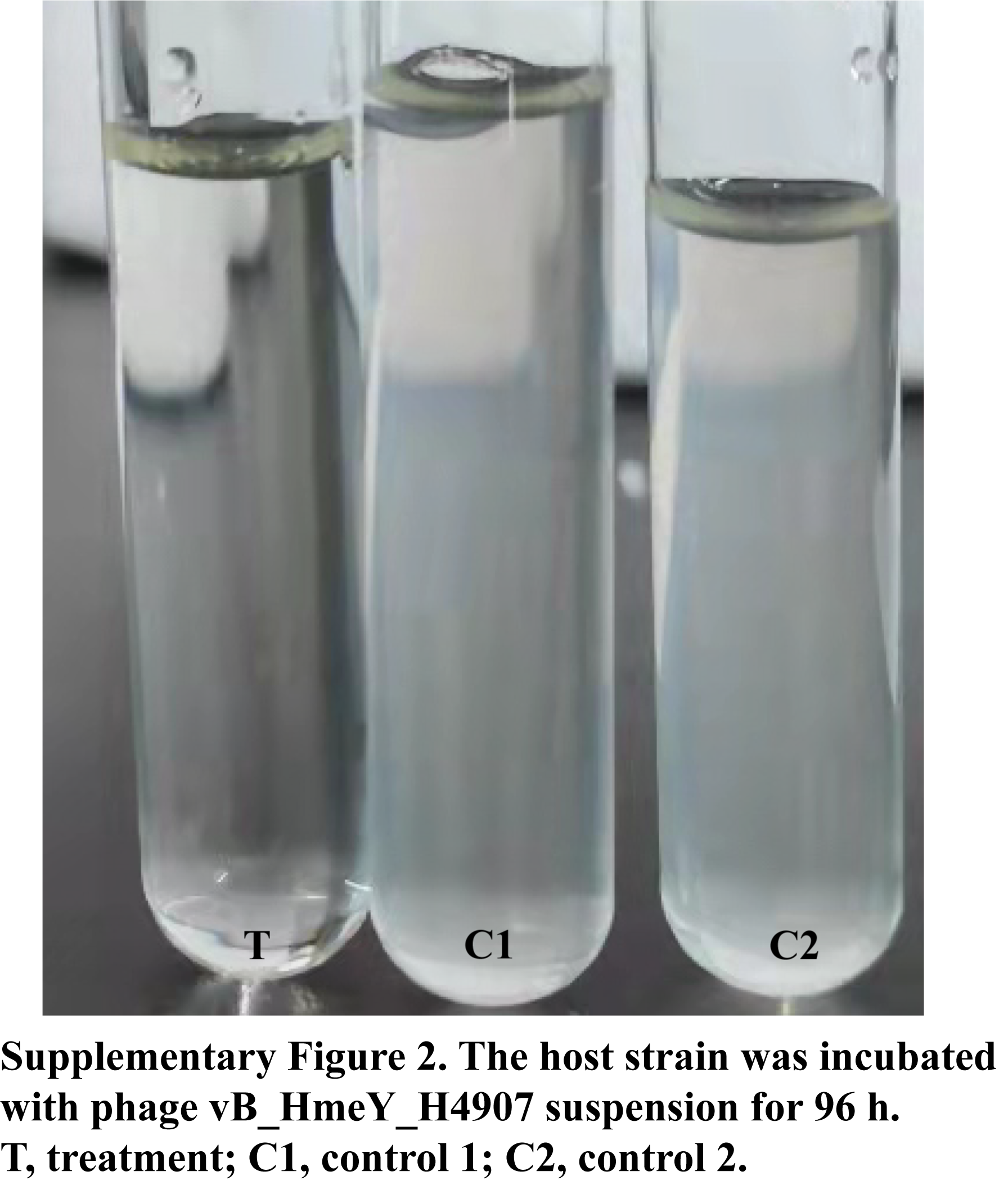

Supplement: Fig. S2 — The host strain was incubated with phage vB_HmeY_H4907 suspension for 96 h. [file spectrum.01912-23-s0002.tif]

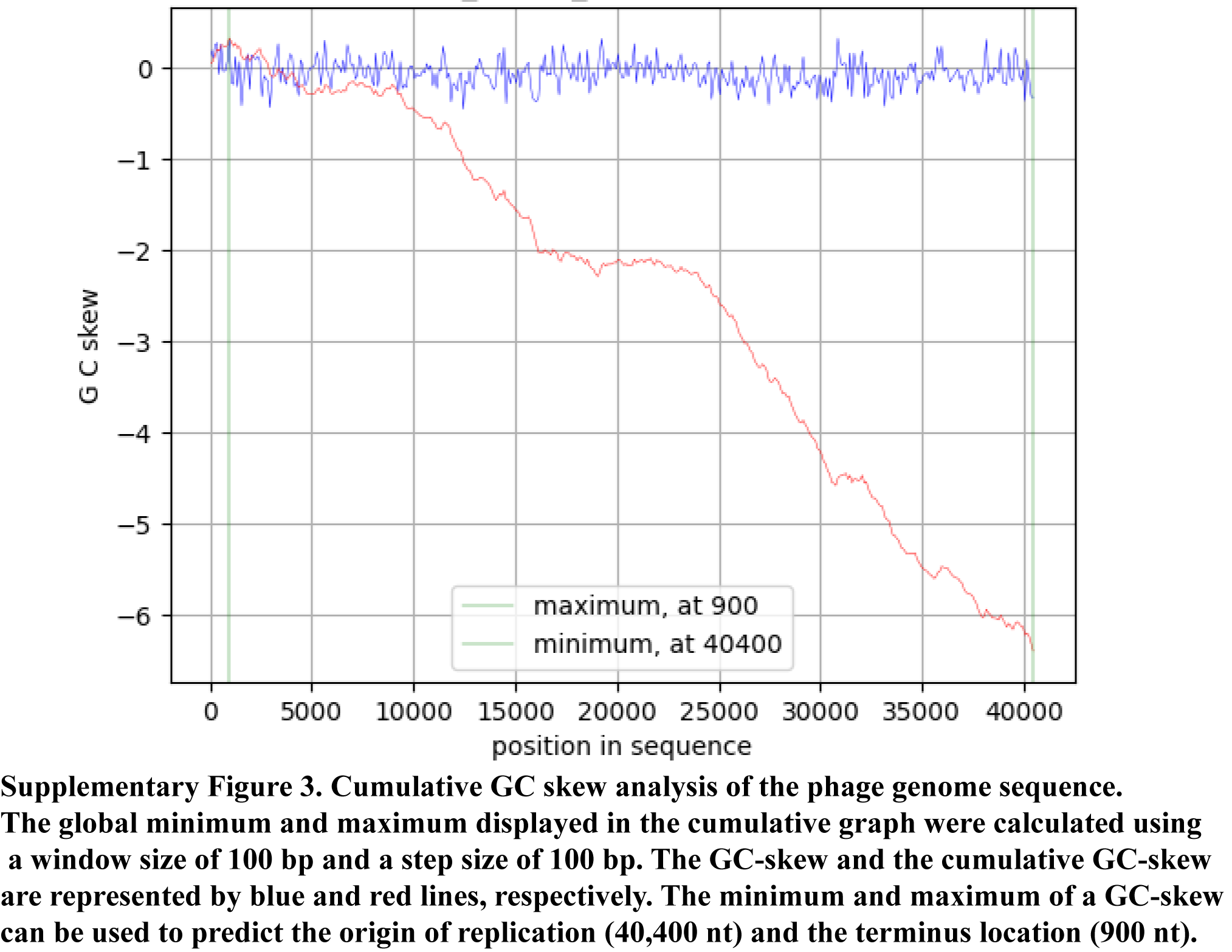

Supplement: Fig. S3 — Cumulative GC skew analysis of the phage genome sequence. [file spectrum.01912-23-s0003.tif]

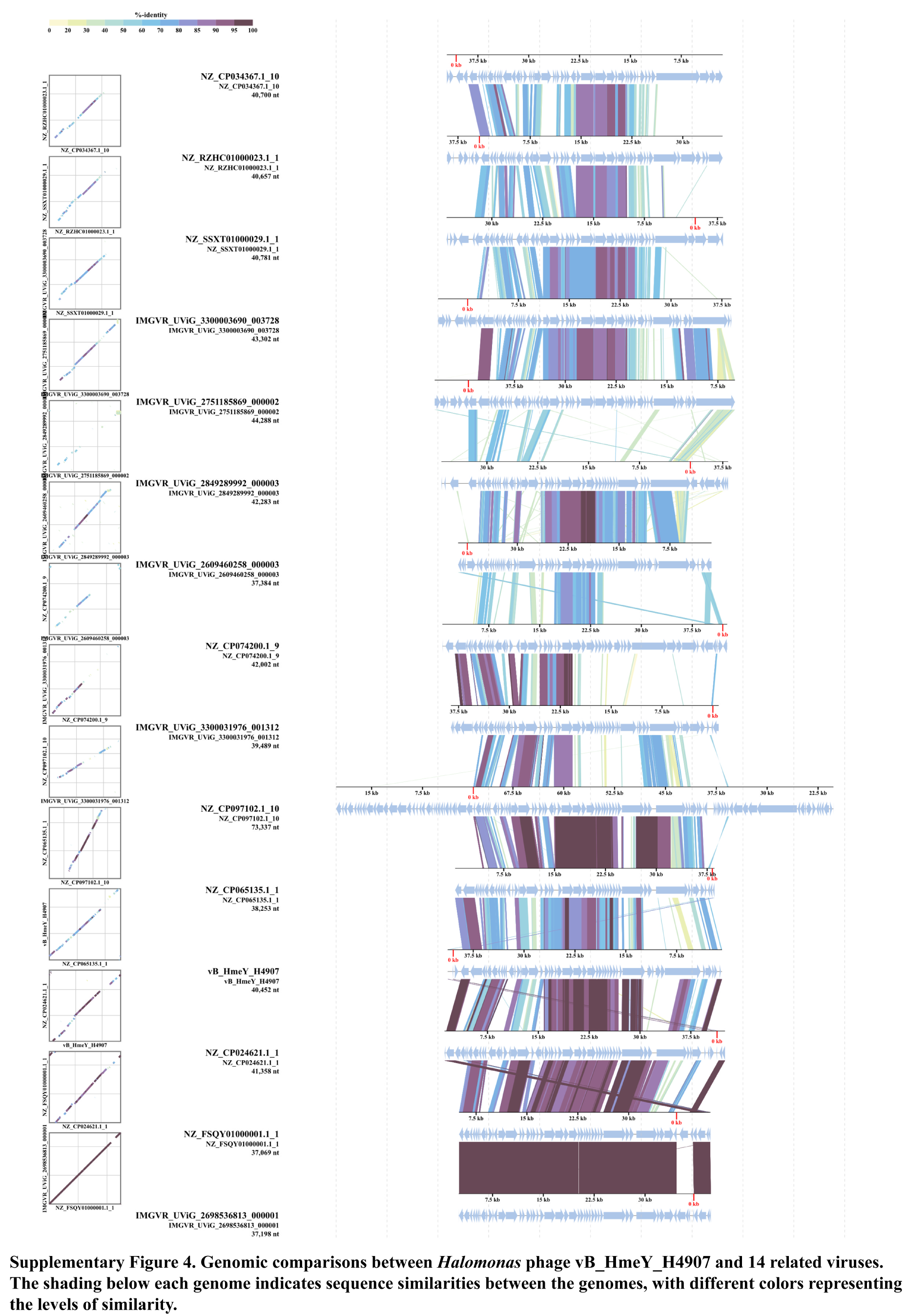

Supplement: Fig. S4 — Genomic comparisons between Halomonas phage vB_HmeY_H4907 and 14 related viruses. [file spectrum.01912-23-s0004.tif]

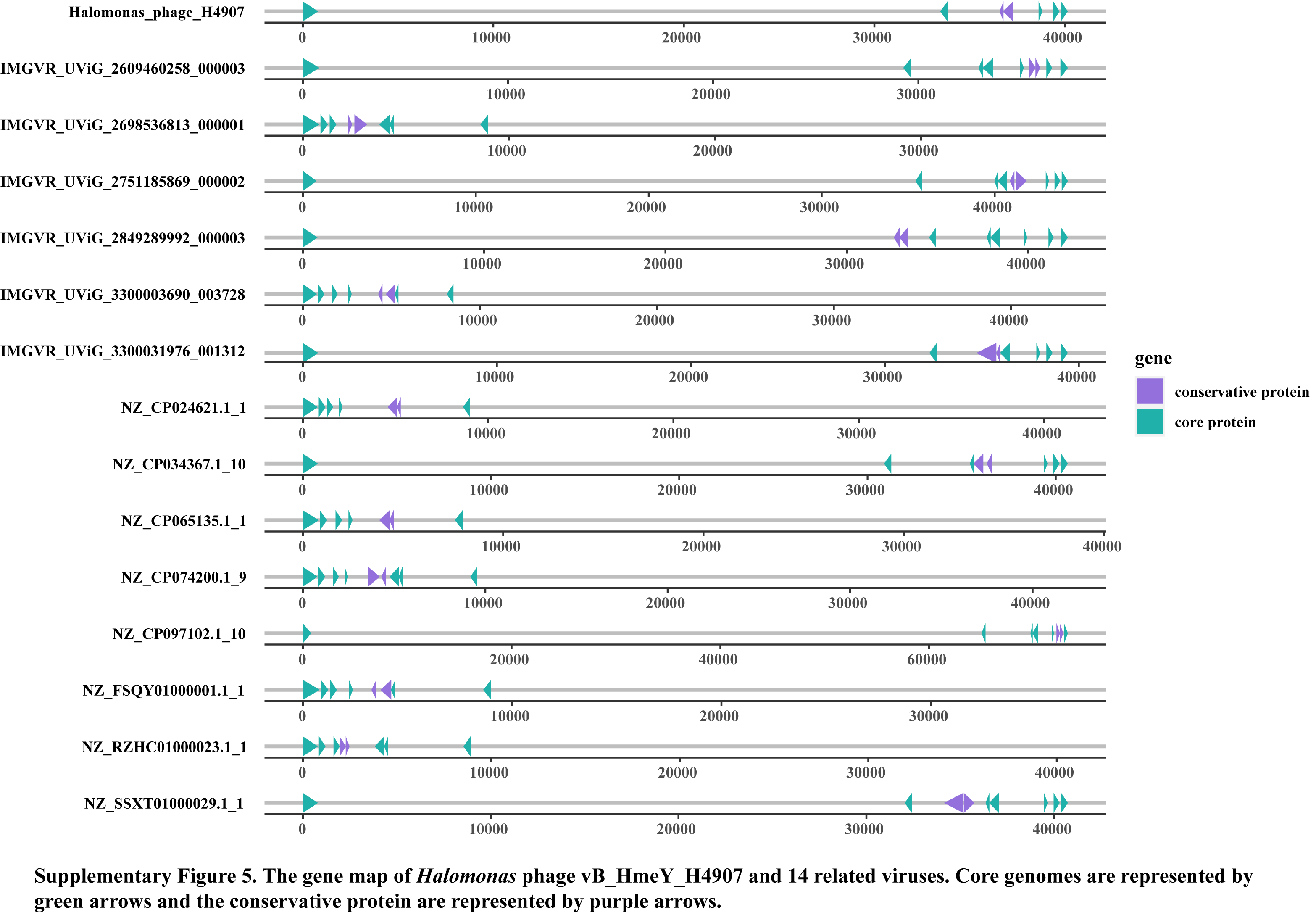

Supplement: Fig. S5 — Gene map of Halomonas phage vB_HmeY_H4907 and 14 related viruses. [file spectrum.01912-23-s0005.tif]

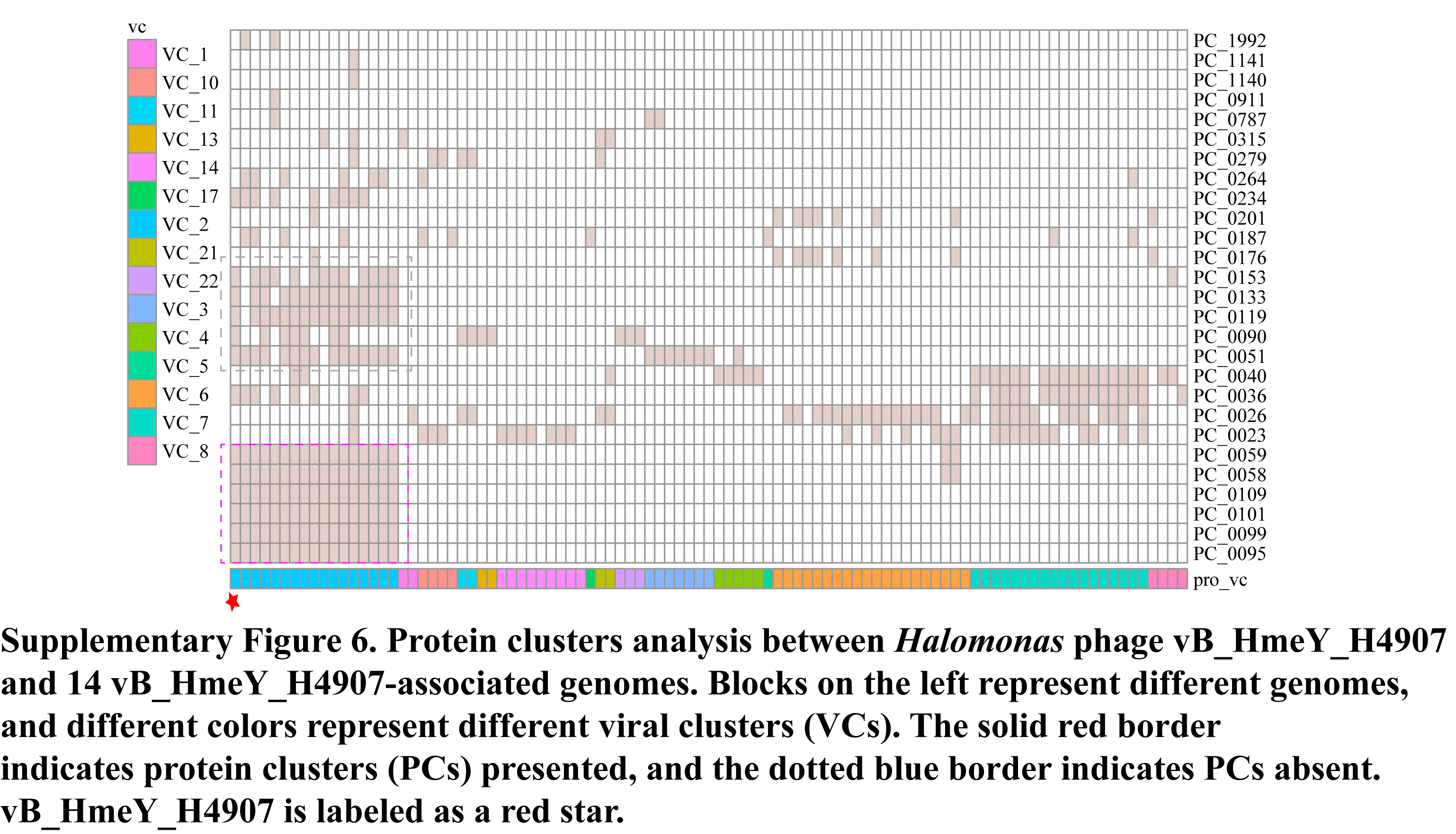

Supplement: Fig. S6 — Protein cluster analysis between Halomonas phage vB_HmeY_H4907 and 14 vB_HmeY_H4907-associated genomes. [file spectrum.01912-23-s0006.tif]
