# Supplementary material for: Identification and genomic analysis of temperate Halomonas bacteriophage vB_HmeY_H4907 from the surface sediment of the Mariana Trench at a depth of 8,900 m
Source: Microbiol Spectr. 2023 Sep 20;11(5):e01912-23. doi: 10.1128/spectrum.01912-23 (PMC10580944; doi:10.1128/spectrum.01912-23)
Supplement: Table S1 — Genome annotation of Halomonas phage vB_HmeY_H4907. [file spectrum.01912-23-s0007.docx]

| **Table S1 Genome annotation of Halomonas phage vB_HmeY_H4907** | | | | | | | | | | | | |  |
| --- | --- | --- | --- | --- | --- | --- | --- | --- | --- | --- | --- | --- | --- |
| **Gene** | **Coding region** | **Length(aa)** | **Strand** | **Start condon** | **Best hits against nr database** | **E value** | **Query coverage (%)** | **aa Identity (%)** | **Bitsocre** | **conserved domain** | **Putative function** | [**module**](file:///C:\Users\Yue%20Su\Desktop\桌面总\4907final\文章\LenovoSoftstore\Install\wangyiyoudaocidian\8.9.6.0\resultui\html\index.html#/javascript:;) | **Methods and databases for searching domins** |
| 1 | 124_213 | 30 | + | ATG |  |  |  |  |  | No hit | Hypothetical protein | Unclassified | |
| 2 | 306_647 | 113 | + | ATG | WP_237673432.1 | 5E-50 | 1 | 69.9 |  | No hit | Hypothetical protein | Unclassified | Methods and databases for searching domins |
| 3 | 967_1260 | 97 | - | ATG | WP_159340776.1 | 2E-63 | 1 | 97.9 |  | No hit | Hypothetical protein | Unclassified | |
| 4 | 1505_1930 | 141 | - | ATG | WP_235253342.1 | 2E-72 | 1 | 83.7 |  | P51736(0.00000000084) | Hypothetical protein | Unclassified | pfam-scan/PfamA |
| 5 | 1927_2244 | 105 | - | ATG | HAO02935.1 | 3E-55 | 0.97 | 86.3 |  | No hit | Hypothetical protein | Unclassified | |
| 6 | 2257_3243 | 328 | - | ATG | WP_211596043.1 | 5E-62 | 0.71 | 50.6 |  | No hit | Hypothetical protein | Unclassified | |
| 7 | 3240_3599 | 119 | - | TTG | WP_228219968.1 | 2E-59 | 1 | 88.2 |  | P51736(0.00000000048) | Hypothetical protein | Unclassified | pfam-scan/PfamA |
| 8 | 3586_3954 | 122 | - | ATG | WP_228219967.1 | 7E-76 | 1 | 95.9 |  | No hit | Hypothetical protein | Unclassified | |
| 9 | 3954_9233 | 1759 | - | ATG | WP_062359637.1 | 0 | 0.93 | 97 | 91.5 | PF13550.8(5E-26) | Putative phage tail protein | Structure and packaging | pfam-scan/PfamA |
| 10 | 9230_9826 | 198 | - | ATG | WP_062359635.1 | 2E-116 | 1 | 100 |  | O64334(1.5E-34) | Tail tip assembly protein | Structure and packaging | Hhpred-UniProtKB |
| 11 | 9823_10590 | 256 | - | ATG | WP_062359633.1 | 8E-169 | 0.93 | 0.96 | 476 | O64333(5E-31) | Tail tip assembly protein | Structure and packaging | Hhpred-UniProtKB |
| 12 | 10646_10879 | 78 | + | ATG | WP_228219717.1 | 5E-157 | 0.93 | 0.87 | 446 | No hit | Hypothetical protein | Unclassified | |
| 13 | 10860_11105 | 81 | - | ATG | WP_062359629.1 | 3E-40 | 0.9 | 91.8 |  | No hit | Hypothetical protein | Unclassified | |
| 14 | 11114_11815 | 233 | - | ATG | WP_228219714.1 | 2E-168 | 1 | 97.4 | 139 | PF05100.14(1.6E-40) | Phage minor tail protein L | Structure and packaging | pfam-scan/PfamA |
| 15 | 11812_12210 | 133 | - | ATG | WP_227390590.1 | 1E-53 | 0.87 | 0.68 | 175 | P03737(2.7E-35) | Tail tip protein M | Structure and packaging | Hhpred-UniProtKB |
| 16 | 12167_15490 | 1107 | - | ATG | WP_159340767.1 | 0 | 0.81 | 94.2 | 155 | PF20155.1(1.5E-45) | Tape measure protein | Structure and packaging | pfam-scan/PfamA |
| 17 | 15546_15884 | 112 | - | ATG |  |  |  |  |  | No hit | Hypothetical protein | Unclassified | |
| 18 | 16118_16408 | 96 | - | ATG | WP_074211116.1 | 3E-60 | 0.97 | 100 | 29.4 | PF06223.15(5E-22) | Minor tail protein | Structure and packaging | pfam-scan/PfamA |
| 19 | 16435_16863 | 142 | - | ATG | WP_159340764.1 | 1E-84 | 1 | 97.9 | 99.1 | PF06222.13(1.6E-28) | Phage tail assembly chaperone | Structure and packaging | pfam-scan/PfamA |
| 20 | 16867_17340 | 157 | - | ATG | WP_206048601.1 | 1E-88 | 0.96 | 96.7 | 31.2 | PF16461.7(0.00000016) | Lambda phage tail tube protein, TTP | Structure and packaging | pfam-scan/PfamA |
| 21 | 17396_17725 | 109 | - | ATG | WP_074211119.1 | 1E-69 | 1 | 98.2 | 38.1 | 6TE9_F(0.00000000000003) | Tail terminator protein | Structure and packaging | Hhpred-PDB |
| 22 | 17722_18141 | 139 | - | GTG | WP_074211120.1 | 1E-75 | 1 | 100 | 35.1 | PF04883.14(0.000000022) | Bacteriophage HK97-gp10, putative tail-component | Structure and packaging | pfam-scan/PfamA |
| 23 | 18138_18458 | 106 | - | ATG | WP_159340761.1 | 1E-71 | 1 | 100 | 79.5 | PF05521.13(2.1E-22) | Phage head-tail joining protein | Structure and packaging | pfam-scan/PfamA |
| 24 | 18455_18985 | 176 | - | ATG | WP_159340760.1 | 4E-109 | 1 | 97.7 |  | 6TE9_C(5.8E-26) | Adaptor protein | Structure and packaging | Hhpred-PDB |
| 25 | 18982_19362 | 126 | - | ATG | WP_159340759.1 | 5E-24 | 1 | 96.8 |  | cd21697(0.59) | Replicative helicase | Nucleotide metabolism | Batch CD-Search/CD |
| 26 | 19425_20672 | 415 | - | ATG | WP_159340758.1 | 0 | 1 | 99.3 | 227 | PF05065.15(2.3E-67) | Major capsid protein | Structure and packaging | pfam-scan/PfamA |
| 27 | 20738_21448 | 236 | - | ATG | WP_235253031.1 | 3E-157 | 1 | 99.2 | 87.9 | PF00574.25(7.8E-25) | ATP-dependent Clp protease | Structure and packaging | pfam-scan/PfamA |
| 28 | 21435_22712 | 425 | - | GTG | WP_217352975.1 | 0 | 0.98 | 97.9 | 273 | PF04860.14(4.6E-81) | Phage portal protein | Structure and packaging | pfam-scan/PfamA |
| 29 | 22724_24445 | 574 | - | ATG | WP_074211125.1 | 0 | 0.99 | 0.99 | 1168 | P59217(1.2E-22) | Terminase large subunit | Structure and packaging | Hhpred-UniProtKB |
| 30 | 24408_24923 | 172 | - | ATG | WP_159340755.1 | 2E-118 | 0.99 | 0.96 | 342 | 6Z6E_A(0.000000000064) | Terminase small subunit | Structure and packaging | Hhpred-PDB |
| 31 | 25139_25459 | 106 | - | ATG | WP_074211127.1 | 3E-73 | 1 | 100 | 35.8 | PF01844.25(0.0000000072) | HNH endonuclease | Nucleotide metabolism | pfam-scan/PfamA |
| 32 | 25595_26026 | 143 | - | ATG | WP_074211128.1 | 3E-95 | 1 | 98.6 |  | P51770(4E-19) | Probable spanin protein | Lytic | Hhpred/UniProtKB |
| 33 | 26023_26346 | 107 | - | ATG | WP_074211129.1 | 2E-70 | 1 | 100 | 82.4 | PF05106.14(2.7E-23) | Phage holin family (Lysis protein S) | Lytic | pfam-scan/PfamA |
| 34 | 26372_26950 | 192 | - | ATG | WP_083602233.1 | 2E-125 | 0.91 | 99.4 | 49.4 | PF01520.20(0.00000000000056) | N-acetylmuramoyl-L-alanine amidase | Nucleotide metabolism | pfam-scan/PfamA |
| 35 | 27210_27587 | 125 | - | ATG | WP_235253025.1 | 9E-85 | 1 | 97.6 |  | Q9T1U3(0.000000000003) | Antitermination protein | Nucleotide metabolism | Hhpred-UniProtKB |
| 36 | 27709_27825 | 39 | + | ATG |  |  |  |  |  | No hit | Hypothetical protein | Unclassified | |
| 37 | 27833_29296 | 487 | - | ATG | WP_235253024.1 | 0 | 1 | 97.3 | 54.4 | PF19263.1(0.00000000000002) | Primase | Nucleotide metabolism | pfam-scan/PfamA |
| 38 | 29296_30324 | 342 | - | ATG | WP_137094363.1 | 0 | 0.99 | 95.3 | 53.7 | PF08707.13(0.000000000000021) | Primase C terminal 2 (PriCT-2) | Nucleotide metabolism | pfam-scan/PfamA |
| 39 | 30324_30524 | 66 | - | GTG | WP_137094371.1 | 8E-37 | 1 | 92.4 | 44.4 | PF01258.19(0.000000000012) | Prokaryotic dksA/traR C4-type zinc finger | Transcriptional regulation | pfam-scan/PfamA |
| 40 | 30517_31026 | 169 | - | ATG | WP_137094373.1 | 2E-114 | 1 | 94.7 |  | PF06892.14(3.5E-20) | Phage regulatory protein | Transcriptional regulation | pfam-scan/PfamA |
| 41 | 31214_31417 | 68 | - | ATG | WP_240568078.1 | 1E-21 | 0.92 | 0.63 | 89.7 | 2O38_B(4.7E-24) | Transcriptional Regulator | Transcriptional regulation | Hhpred-PDB |
| 42 | 31529_32266 | 245 | + | ATG | WP_127042724.1 | 3E-130 | 0.95 | 76.2 | 67.3 | PF00717.25(0.000000000000000001) | Peptidase S24-like | Nucleotide metabolism | pfam-scan/PfamA |
| 43 | 32373_32564 | 63 | + | ATG | WP_074211139.1 | 1E-21 | 1 | 98.4 |  | No hit | Hypothetical protein | Unclassified | |
| 44 | 32561_32821 | 86 | + | ATG | WP_074211140.1 | 1E-53 | 1 | 97.7 |  | No hit | Hypothetical protein | Unclassified | |
| 45 | 32818_33144 | 108 | + | ATG | WP_074211141.1 | 7E-72 | 1 | 99.1 |  | No hit | Hypothetical protein | Unclassified | |
| 46 | 33141_33461 | 106 | + | ATG | WP_137094384.1 | 2E-68 | 1 | 99.1 | 36.6 | PF03374.16(0.0000000049) | Phage antirepressor protein KilAC domain | Transcriptional regulation | pfam-scan/PfamA |
| 47 | 33544_33903 | 119 | + | ATG | WP_235253019.1 | 6E-81 | 1 | 98.3 | 244 | PF10065.11(1.2E-72) | Uncharacterized conserved protein (DUF2303) | Unclassified | pfam-scan/PfamA |
| 48 | 33961_34764 | 268 | + | ATG | TKJ10232.1 | 0 | 99% | #### | 541 | PF10065.12 | Uncharacterized conserved protein(1.6E-19) | Unclassified | pfam-scan/PfamA |
| 49 | 34836_35483 | 215 | + | ATG | HBK35805.1 | 1E-33 | 1 | 96.7 | 445 | No hit | Hypothetical protein | Unclassified | |
| 50 | 35480_35725 | 81 | + | ATG |  |  |  |  |  | No hit | Hypothetical protein | Unclassified | |
| 51 | 35722_36216 | 164 | + | ATG | WP_159340737.1 | 1E-33 | 1 | 100 | 139 | No hit | Hypothetical protein | Unclassified | |
| 52 | 36213_37931 | 572 | + | ATG | WP_235253014.1 | 0 | 99% | #### | 1165 | P09915 | Modification methylase | Transcriptional regulation | Hhpred-UniProtKB |
| 53 | 38177_39337 | 386 | + | GTG | WP_159340731.1 | 0 | 0.99 | 96.9 | 81 | PF12167.10(4.7E-23) | Arm DNA-binding domain | Transcriptional regulation | pfam-scan/PfamA |
| 54 | 39327_39719 | 130 | - | GTG | WP_232862134.1 | 8E-24 | 0.83 | 46.3 |  | No hit | Hypothetical protein | Unclassified | |
| 55 | 39728_40213 | 161 | - | ATG | WP_047585881.1 | 3E-62 | 0.98 | 59.1 | 74.8 | PF13274.8(1E-20) | Protein of unknown function (DUF4065) | Unclassified | pfam-scan/PfamA |
