# Supplementary material for: Identification and genomic analysis of temperate Halomonas bacteriophage vB_HmeY_H4907 from the surface sediment of the Mariana Trench at a depth of 8,900 m
Source: Microbiol Spectr. 2023 Sep 20;11(5):e01912-23. doi: 10.1128/spectrum.01912-23 (PMC10580944; doi:10.1128/spectrum.01912-23)
Supplement: Table S2 — HHperd hits of Halomonas phage vB_HmeY_H4907. [file spectrum.01912-23-s0008.docx]

| **Table S2 HHperd hits of Halomonas phage vB_HmeY_H4907** | | | | | | | | | |
| --- | --- | --- | --- | --- | --- | --- | --- | --- | --- |
| **Gene** | **Coding region** | **Hit accession** | **Description** | **Probability** | **E-value** | **Score** | **SS** | **Aligned cols** | **Target length** |
| 4 | 1505_1930 | P51736 | YO32_BPHC1 Uncharacterized 23.3 kDa protein in lys 3'region OS=Haemophilus phage HP1 (strain HP1c1) OX=1289570 PE=4 SV=1 | 99.2 | 8.4E-10 | 76.3 | 12 | 125 | 200 |
| 7 | 3240_3599 | P51736 | YO32_BPHC1 Uncharacterized 23.3 kDa protein in lys 3'region OS=Haemophilus phage HP1 (strain HP1c1) OX=1289570 PE=4 SV=1 | 99.3 | 4.8E-10 | 80.3 | 15 | 109 | 200 |
| 9 | 3954_9233 | Q6QGE9 | BPPB3_BPT5 Probable baseplate hub protein OS=Escherichia phage T5 OX=10726 GN=D16 PE=2 SV=1 | 100 | 5.4E-40 | 461 | 58 | 490 | 949 |
| 10 | 9230_9826 | O64334 | TIPI_BPN15 Tail tip assembly protein I OS=Escherichia phage N15 OX=40631 GN=gene 20 PE=3 SV=1 | 100 | 1.5E-34 | 221 | 23 | 189 | 192 |
| 11 | 9823_10590 | O64333 | TIPK_BPN15 Tail tip assembly protein K OS=Escherichia phage N15 OX=40631 GN=gene 19 PE=3 SV=1 | 100 | 5E-31 | 212 | 28 | 226 | 243 |
| 14 | 11114_11815 | O64332 | TIPL_BPN15 Tail tip protein L OS=Escherichia phage N15 OX=40631 GN=gene 18 PE=3 SV=1 | 100 | 2.7E-35 | 239 | 24 | 219 | 251 |
| 15 | 11812_12210 | P03737 | TIPM_LAMBD Tail tip protein M OS=Escherichia phage lambda OX=10710 GN=M PE=3 SV=1 | 99.9 | 1.2E-22 | 128 | 13 | 101 | 109 |
| 16 | 12167_15490 | Q6XQC4 | TMP_BPT1 Tape measure protein OS=Escherichia phage T1 OX=1921008 GN=38 PE=3 SV=1 | 99.8 | 6.4E-11 | 156 | 95 | 136 | 957 |
| 18 | 16118_16408 | PF06223.15 | ; Phage_tail_T ; Minor tail protein T | 99.9 | 5E-22 | 126 | 10 | 87 | 99 |
| 19 | 16435_16863 | 2OB9_A | Tail assembly chaperone; Bacteriophage HK97, morphogenesis, tail assembly chaperone, CHAPERONE; HET: MSE; 2.3A {Enteroba | 99.9 | 4.7E-24 | 148 | 12 | 128 | 130 |
| 20 | 16867_17340 | P85503 | STRU3_BPPAJ Structural protein 3 OS=Pseudomonas phage PAJU2 OX=504346 PE=1 SV=2 | 99.9 | 1.6E-19 | 125 | 18 | 141 | 164 |
| 21 | 17396_17725 | 6TE9_F | Tail terminator protein Rcc01690; "neck", "portal", "capsid", "tail tube", VIRUS; 3.58A {Rhodobacter capsulatus} | 99.6 | 3E-14 | 81 | 11 | 105 | 135 |
| 22 | 17722_18141 | PF11114.11 | ; Minor_capsid_2 ; Minor capsid protein | 99.4 | 2.6E-12 | 74.3 | 4 | 81 | 105 |
| 23 | 18138_18458 | 2KZ4_A | Putative head-tail adaptor; Structural Genomics, PSI-2, Protein Structure Initiative, Northeast Structural Genomics Cons | 99.9 | 5.8E-22 | 107 | 14 | 105 | 112 |
| 24 | 18455_18985 | 6TE9_C | Adaptor protein Rcc01688; "neck", "portal", "capsid", "tail tube", VIRUS; 3.58A {Rhodobacter capsulatus} | 100 | 5.8E-26 | 167 | 20 | 173 | 197 |
| 25 | 18982_19362 | cd21697 | GINS_B_archaea_Gins23; beta-strand (B) domain of archaeal GINS complex protein Gins23. The GINS (named from the Japanese | 91.7 | 0.59 | 31.2 | 3 | 36 | 42 |
| 26 | 19425_20672 | 3QPR_D | Major capsid protein; Virus Procapsid particles, VIRUS; 5.2A {Enterobacteria phage HK97} | 100 | 1.5E-37 | 292 | 25 | 374 | 385 |
| 27 | 20738_21448 | 7EKO_G | ATP-dependent Clp protease proteolytic subunit; Clp, Complex, Protease, Chloroplast, Chlamydomnas, Atp-dependent., STRUC | 99.9 | 1.7E-19 | 145 | 19 | 179 | 296 |
| 28 | 21435_22712 | P49859 | PORTL_BPHK7 Portal protein OS=Enterobacteria phage HK97 OX=37554 GN=3 PE=3 SV=1 | 100 | 8.2E-43 | 339 | 47 | 356 | 424 |
| 29 | 22724_24445 | P59217 | TERL_BPSF5 Putative terminase large subunit OS=Shigella phage SfV OX=55884 GN=2 PE=3 SV=1 | 100 | 2.4E-61 | 507 | 68 | 540 | 577 |
| 30 | 24408_24923 | 6Z6E_A | Terminase small subunit; genome packaging, bacteriophage, DNA binding, VIRAL PROTEIN; 1.4A {Enterobacteria phage HK97} | 99.7 | 3.8E-17 | 118 | 10 | 103 | 160 |
| 31 | 25139_25459 | 5H0M_A | HNH endonuclease; Thermophilic bacteriophage, HNH Endonuclease, DNA nicking, HYDROLASE; 1.52A {Geobacillus virus E2} | 97.6 | 0.00038 | 38.8 | 4 | 65 | 130 |
| 32 | 25595_26026 | P51770 | SPAN1_BPP2 Probable spanin, inner membrane subunit OS=Escherichia phage P2 OX=10679 GN=lysB PE=3 SV=1 | 99.9 | 4E-19 | 125 | 18 | 134 | 141 |
| 33 | 26023_26346 | P03705 | HOLIN_LAMBD Antiholin OS=Escherichia phage lambda OX=10710 GN=S PE=1 SV=1 | 99.9 | 1.3E-23 | 130 | 15 | 103 | 107 |
| 34 | 26372_26950 | 4BIN_A | N-ACETYLMURAMOYL-L-ALANINE AMIDASE AMIC; HYDROLASE, BACTERIAL DIVISION; 2.49A {ESCHERICHIA COLI} | 99.7 | 8.3E-13 | 92.3 | 23 | 167 | 403 |
| 35 | 27210_27587 | Q9T1U3 | REGQ_BPAPS Probable antitermination protein Q OS=Acyrthosiphon pisum secondary endosymbiont phage 1 OX=67571 GN=5 PE=3 S | 99.5 | 3E-12 | 78.5 | 13 | 111 | 148 |
| 37 | 27833_29296 | 6K9C_B | Primase; primase, helicase, ssDNA-binding protein, TRANSFERASE; HET: SO4; 2.406A {Nitratiruptor phage NrS-1} | 100 | 8.60E-26 | 225 | 32 | 313 | 416 |
| 37 | 27833_29296 | 6K9C_A | Primase; primase, helicase, ssDNA-binding protein, TRANSFERASE; HET: SO4; 2.406A {Nitratiruptor phage NrS-1} | 100 | 8.2E-30 | 254 | 34 | 303 | 416 |
| 38 | 29296_30324 | P10277 | PRIM_BPP4 Putative P4-specific DNA primase OS=Enterobacteria phage P4 OX=10680 GN=Alpha PE=1 SV=1 | 99.9 | 1.9E-20 | 178 | 13 | 251 | 777 |
| 39 | 30324_30524 | Q06424 | YO82_BPP2 Uncharacterized 8.2 kDa protein in gpA 5'region OS=Escherichia phage P2 OX=10679 GN=ORF82 PE=4 SV=1 | 99.8 | 5.5E-20 | 91.2 | 7 | 64 | 74 |
| 40 | 30517_31026 | PF06892.14 | ; Phage_CP76 ; Phage regulatory protein CII (CP76) | 99.9 | 3.5E-20 | 128 | 19 | 139 | 142 |
| 41 | 31214_31417 | 2O38_B | Hypothetical protein; alpha-beta, helix-turn-helix, Structural Genomics, PSI-2, Protein Structure Initiative, Midwest Ce | 98 | 0.000018 | 39.9 | 2 | 40 | 120 |
| 42 | 31529_32266 | P03034 | RPC1_LAMBD Repressor protein cI OS=Escherichia phage lambda OX=10710 GN=cI PE=1 SV=2 | 99.9 | 2.2E-23 | 160 | 20 | 213 | 237 |
| 48 | 33961_34764 | PF10065.12 | DUF2303 ; Uncharacterized conserved protein (DUF2303) | 100 | 1.4E-56 | 400 | 30 | 257 | 269 |
| 52 | 36213_37931 | P09915 | MTBR_BPRH1 Modification methylase Rho11sI OS=Bacillus phage rho11s OX=10735 PE=3 SV=2 | 100 | 2.80E-40 | 360 | 30 | 169 | 503 |
| 53 | 38177_39337 | P06155 | VINT_BPPH8 Integrase OS=Enterobacteria phage phi80 OX=10713 GN=int PE=3 SV=1 | 100 | 2.2E-35 | 256 | 31 | 354 | 402 |
| 55 | 39728_40213 | PF13274.9 | ; DUF4065 ; Protein of unknown function (DUF4065) | 99.5 | 7.3E-14 | 89.1 | 6 | 95 | 103 |
