# Supplementary material for: Identification and genomic analysis of temperate Halomonas bacteriophage vB_HmeY_H4907 from the surface sediment of the Mariana Trench at a depth of 8,900 m
Source: Microbiol Spectr. 2023 Sep 20;11(5):e01912-23. doi: 10.1128/spectrum.01912-23 (PMC10580944; doi:10.1128/spectrum.01912-23)
Supplement: Table S3 — Information of Yueviridae virus in the IMG/VR database. [file spectrum.01912-23-s0009.docx]

| **Table S3 Information of Yueviridae virus in the IMG/VR database** | | | | | |
| --- | --- | --- | --- | --- | --- |
| **Contig** | **Length** | **Taxonomic classification** | **Sequence origin (doi)** | **MIUViG quality** | **Host taxonomy** |
| IMGVR_UViG_2609460258_000003 | 37384 | r__Duplodnaviria;k__Heunggongvirae;p__Uroviricota;c__Caudoviricetes;;;; | (10.5281/zenodo.7015982) | High-confidence | d__Bacteria;p__Proteobacteria;c__Gammaproteobacteria;o__Oceanospirillales;f__Halomonadaceae;g__Salinicola;s__Salinicola |
| IMGVR_UViG_2698536813_000001 | 37198 | r__Duplodnaviria;k__Heunggongvirae;p__Uroviricota;c__Caudoviricetes;;;; | (10.5281/zenodo.7015982) | High-confidence | d__Bacteria;p__Proteobacteria;c__Gammaproteobacteria;o__Oceanospirillales;f__Halomonadaceae;g__Halomonas;s__Halomonas |
| IMGVR_UViG_2751185869_000002 | 44288 | r__Duplodnaviria;k__Heunggongvirae;p__Uroviricota;c__Caudoviricetes;;;; | (10.5281/zenodo.7015982) | High-confidence | d__Bacteria;p__Proteobacteria;c__Gammaproteobacteria;o__Oceanospirillales;f__Halomonadaceae;g__Halomonas;s__Halomonas |
| IMGVR_UViG_2849289992_000003 | 42283 | r__Duplodnaviria;k__Heunggongvirae;p__Uroviricota;c__Caudoviricetes;;;; | (10.5281/zenodo.7015982) | High-confidence | d__Bacteria;p__Proteobacteria;c__Gammaproteobacteria;o__Oceanospirillales;f__Halomonadaceae;g__Salinicola;s__Salinicola |
| IMGVR_UViG_3300003690_003728 | 43302 | r__Duplodnaviria;k__Heunggongvirae;p__Uroviricota;c__Caudoviricetes;;;; | (10.5281/zenodo.7015982) | High-confidence | d__Bacteria;p__Proteobacteria;c__Gammaproteobacteria;o__Oceanospirillales;f__Halomonadaceae;g__Halomonas; |
| IMGVR_UViG_3300031976_001312 | 39489 | r__Duplodnaviria;k__Heunggongvirae;p__Uroviricota;c__Caudoviricetes;;;; | (10.5281/zenodo.7015982) | High-confidence | ;;;;;; |
