# Supplementary material for: Identification and genomic analysis of temperate Halomonas bacteriophage vB_HmeY_H4907 from the surface sediment of the Mariana Trench at a depth of 8,900 m
Source: Microbiol Spectr. 2023 Sep 20;11(5):e01912-23. doi: 10.1128/spectrum.01912-23 (PMC10580944; doi:10.1128/spectrum.01912-23)
Supplement: Table S4 — Information of provirus in Yueviridae by CheckV predicted in the host sequence associated with Halomonas phage vB_HmeY_H4907. [file spectrum.01912-23-s0010.docx]

**Table S4 Information of provirus in Yueviridae by CheckV predicted in the host sequence associated with Halomonas phage vB_HmeY_H4907.**

| **Contig** | **Length** | **Taxonomic classification** | **checkv_quality** | **Host taxonomy** | **Host Length** | **Provirus at host genomic site** |
| --- | --- | --- | --- | --- | --- | --- |
| NZ_FSQY01000001.1_1 | 37069 | unclassified | High-quality | d__Bacteria;p__Proteobacteria;c__Gammaproteobacteria;o__Oceanospirillales;f__Halomonadaceae;g__Halomonas;s__Halomonas | 3,685,191 bp linear DNA | 2178470-2215538/3685191 |
| NZ_CP034367.1_10 | 40700 | unclassified | Medium-quality | d__Bacteria;p__Proteobacteria;c__Gammaproteobacteria;o__Oceanospirillales;f__Halomonadaceae;g__Halomonas;s__Halomonas | 4,446,698 bp circular DNA | 2172153-2212852/4446698 |
| NZ_RZHC01000023.1_1 | 40657 | unclassified | Medium-quality | d__Bacteria;p__Proteobacteria;c__Gammaproteobacteria;o__Oceanospirillales;f__Halomonadaceae;g__Halomonas;s__Halomonas | 493,092 bp linear DNA | 195343-235999/493092 |
| NZ_SSXT01000029.1_1 | 40781 | unclassified | Medium-quality | d__Bacteria;p__Proteobacteria;c__Gammaproteobacteria;o__Oceanospirillales;f__Halomonadaceae;g__Halomonas;s__Halomonas | 54,193 bp linear DNA | 1-40781/54193 |
| NZ_CP024621.1_1 | 41358 | unclassified | High-quality | d__Bacteria;p__Proteobacteria;c__Gammaproteobacteria;o__Oceanospirillales;f__Halomonadaceae;g__Halomonas;s__Halomonas | 3,860,077 bp circular DNA | 292410-333767/3860077 |
| NZ_CP065135.1_1 | 38253 | unclassified | High-quality | d__Bacteria;p__Proteobacteria;c__Gammaproteobacteria;o__Oceanospirillales;f__Halomonadaceae;g__Halomonas;s__Halomonas | 4,437,754 bp circular DNA | 306994-345246/4437754 |
| NZ_CP074200.1_9 | 42002 | unclassified | High-quality | d__Bacteria;p__Proteobacteria;c__Gammaproteobacteria;o__Oceanospirillales;f__Halomonadaceae;g__Halomonas;s__Halomonas | 4,793,405 bp circular DNA | 4617411-4659412/4793405 |
| NZ_CP097102.1_10 | 73337 | unclassified | High-quality | d__Bacteria;p__Proteobacteria;c__Gammaproteobacteria;o__Oceanospirillales;f__Halomonadaceae;g__Halomonas;s__Halomonas | 4,519,177 bp circular DNA | 2235518-2308854/4519177 |
